# Supplementary material for: Automatic Ventriculomegaly Detection in Fetal Brain MRI: A Step-by-Step Deep Learning Model for Novel 2D-3D Linear Measurements
Source: Diagnostics (Basel). 2023 Jul 13;13(14):2355. doi: 10.3390/diagnostics13142355 (PMC10378043; doi:10.3390/diagnostics13142355)
Supplement: Supplementary file 1 [file diagnostics-13-02355-s001.zip › diagnostics-2480993-supplementary.pdf]

Supplement for the paper:

"Automatic Ventriculomegaly Detection in Fetal Brain MRI; A Step-by-Step Deep Learning Model for Novel 2D-3D Linear Measurements"

Dear Ms. Dejana Janicijevic
Special Issue Editor; Machine Learning and Artificial Intelligence in "Diagnostics" journal.
E-Mail: dennis.zhu@mdpi.com

We would like to submit a **supplement** for the manuscript (Manuscript ID: diagnostics-2480993) entitled "Automatic Ventriculomegaly Detection in Fetal Brain MRI; A Step-by-Step Deep Learning Model for Novel 2D-3D Linear Measurements," which already is accepted in the "Diagnostics" Journal (ISSN 2075-4418). The supplement contains additional figures and a section discussing the challenges and solutions related to Fetal Brain MRI reconstruction.

We believe that the supplement adds significant value to the main paper by providing additional visualizations and insights into the specific aspects of our research. We kindly request the inclusion of this supplement alongside the main paper.

Thank you for considering our supplement submission. We look forward to your feedback and the possibility of its inclusion in the publication.

Sincerely,
Farzan Vahedifard, MD

Research Fellow; Department of Diagnostic Radiology and Nuclear Medicine, Rush University Medical Center | Cell: 312-513-6325; Office: 312-942-3408

Supplement Figure S1 illustrates the step of detecting the largest ventricle at the level of Deep gm, providing a visual representation of the process described in the main paper. Supplement Figure S2 highlights an accuracy problem in segmentation, specifically the black pixels on the periphery of the left ventricle. This figure emphasizes the need for further improvement in the segmentation process.

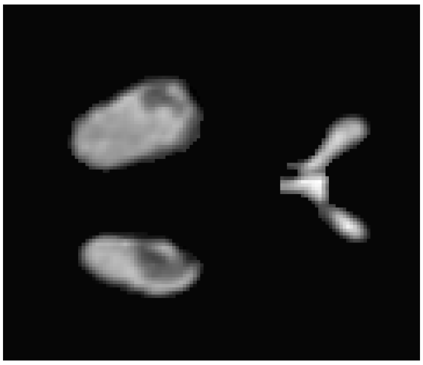

Supplement Figure S1 - Step 7-0: Detection of the largest ventricle, in the level of Deep gm

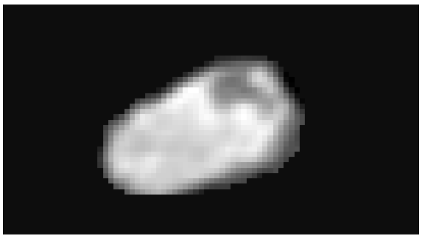

Supplement Figure S2 - black pixels on the left ventricle's periphery: accuracy problem of segmentation

Supplement Figures S3 and S4 present boxplots comparing AI, radiologists, and neuroradiologists' measurements of the Fetal Brain MRI right and left ventricles. These figures offer a comprehensive analysis of the measurements performed by different observers, providing valuable insights into the performance of the AI model in comparison to human experts.

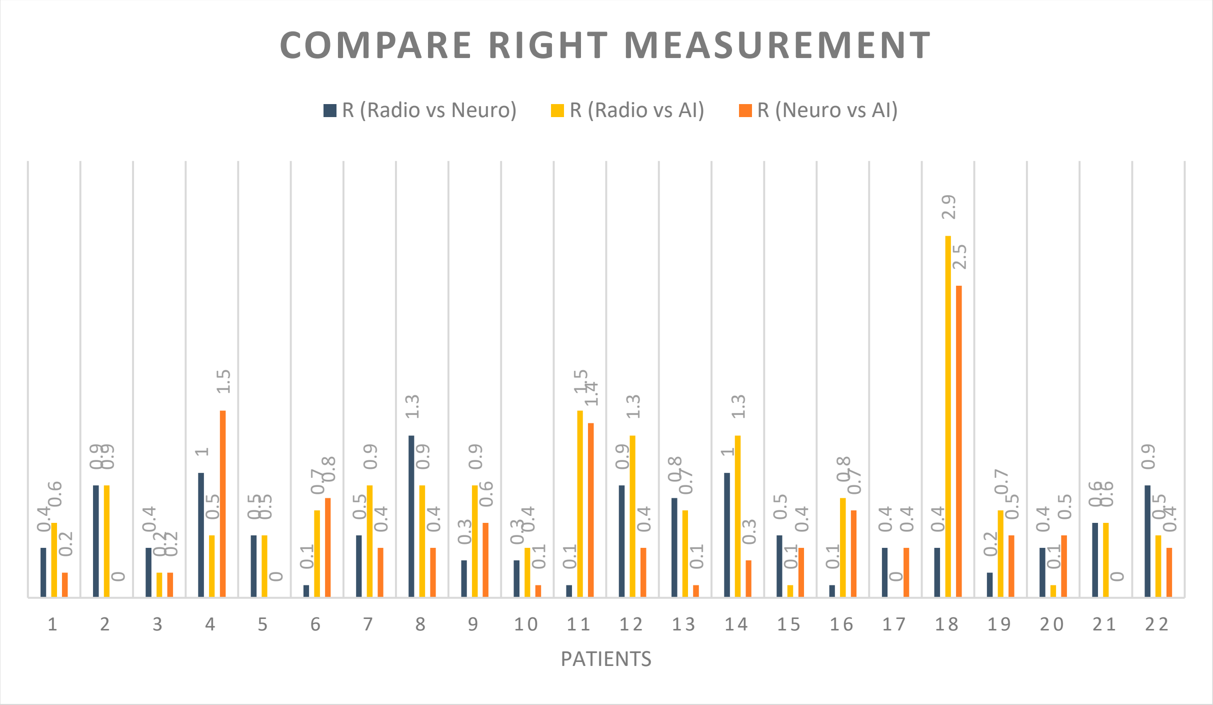

Supplement Figure S3 - Boxplot for comparing the Fetal Brain MRI Right ventricle's measurement by AI, radiologist, and neuroradiologist

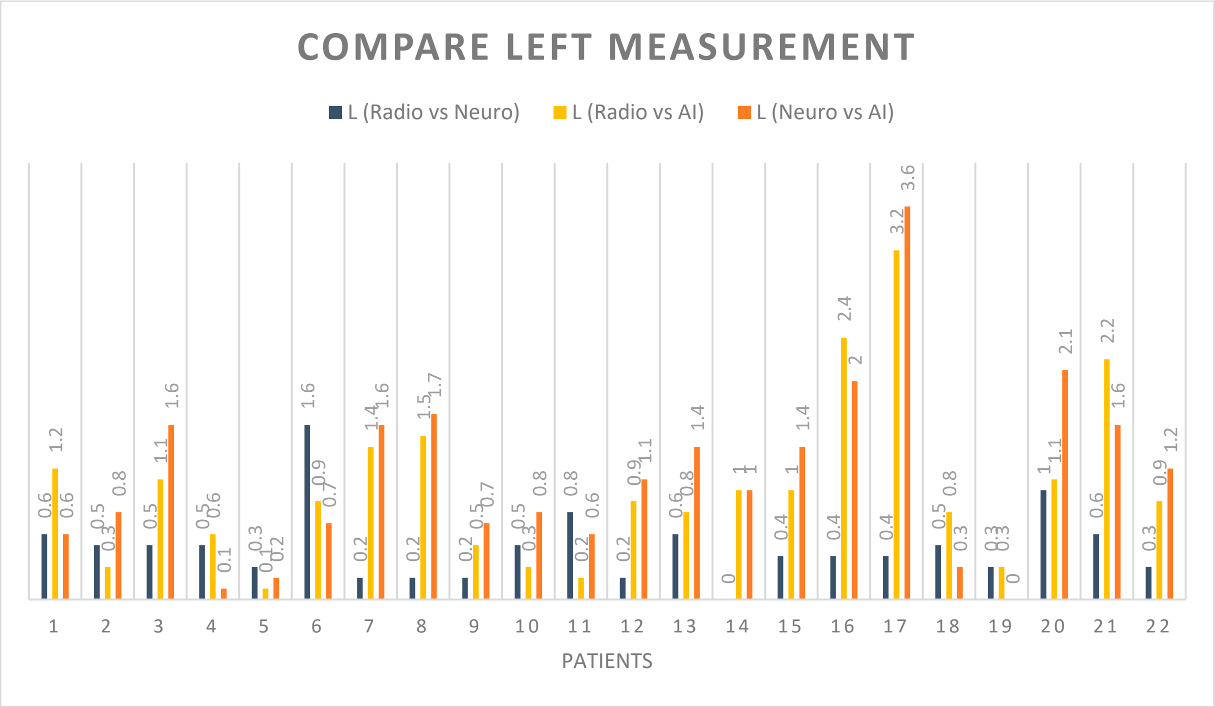

Supplement Figure S4- Boxplot for comparing the Fetal Brain MRI Left ventricle's measurement by AI, radiologist, and neuroradiologist

This supplement section discussing the challenges and solutions related to Fetal Brain MRI reconstruction. We explored various options for selecting suitable cuts for reconstruction and tested different input stacks. The results and implications of these experiments are thoroughly discussed, providing valuable information for researchers and practitioners in the field.

- Supplement section 1: Fetal Brain MRI Reconstruction Challenges and solution:

This supplement details our approach to addressing the challenge of selecting suitable cuts for Fetal Brain MRI Reconstruction. We explored various options and tested different input stacks for the reconstruction process. The following configurations were considered:

- Option 1: Nine input stacks consisting of three axial, three coronal, and three sagittal series.
- Option 2: Five input stacks consisting of three axial, one coronal, and one sagittal series.
- Option 3: Five input stacks consisting of five axial series.

The choice of the number and type of input stacks directly affects the reconstruction time. Since our study focuses on measuring the Ventricle, a structure typically assessed using axial images, we prioritized including as many axial series as possible. In cases where the original dataset had fewer than one or two axial series, the reconstruction stack was adjusted accordingly.

Option 1 for selecting cuts:

As an example, for patient exam_000001, we employed Option 1 for selecting cuts (Supplement Figure S5). This involved selecting the 35/47/44 axial series, the 34/46/36

sagittal series, and the 42/45/50 coronal series. The reconstruction process took approximately 42 minutes, and the results are summarized as follows:

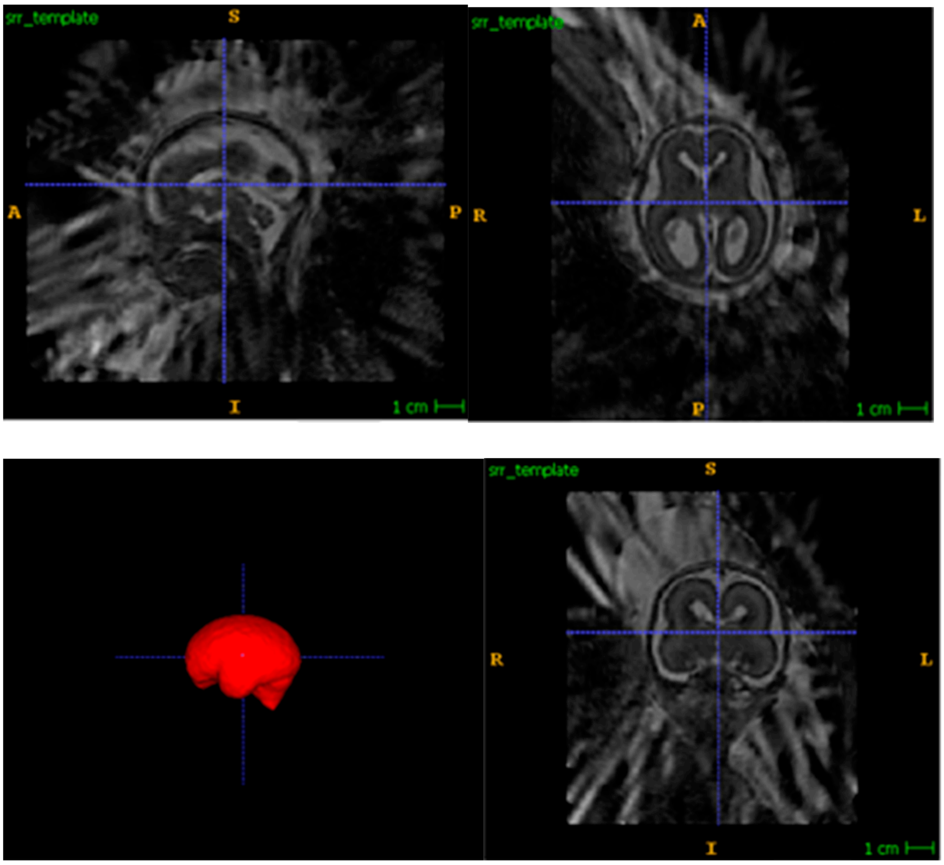

Supplement Figure S5 - Option 1 for selecting cuts for 3D reconstruction

• Option 2 for selecting cuts:

We selected the 35/47/44 axial series, the 34 sagittal series, and the 42 coronal series for this configuration (Supplement Figure S6). The reconstruction process for these cuts took approximately 22 minutes. The results of this reconstruction are as follows:

- Compared to using 9 stacks, we saved a significant time of 20 minutes with nearly the same axial image quality. However, it should be noted that the sagittal and coronal image quality was slightly compromised in this configuration. Nevertheless, since our focus was on measuring the Ventricle, which primarily relies on axial images, the

reduced quality in sagittal and coronal views was acceptable as they were not crucial for our specific analysis.

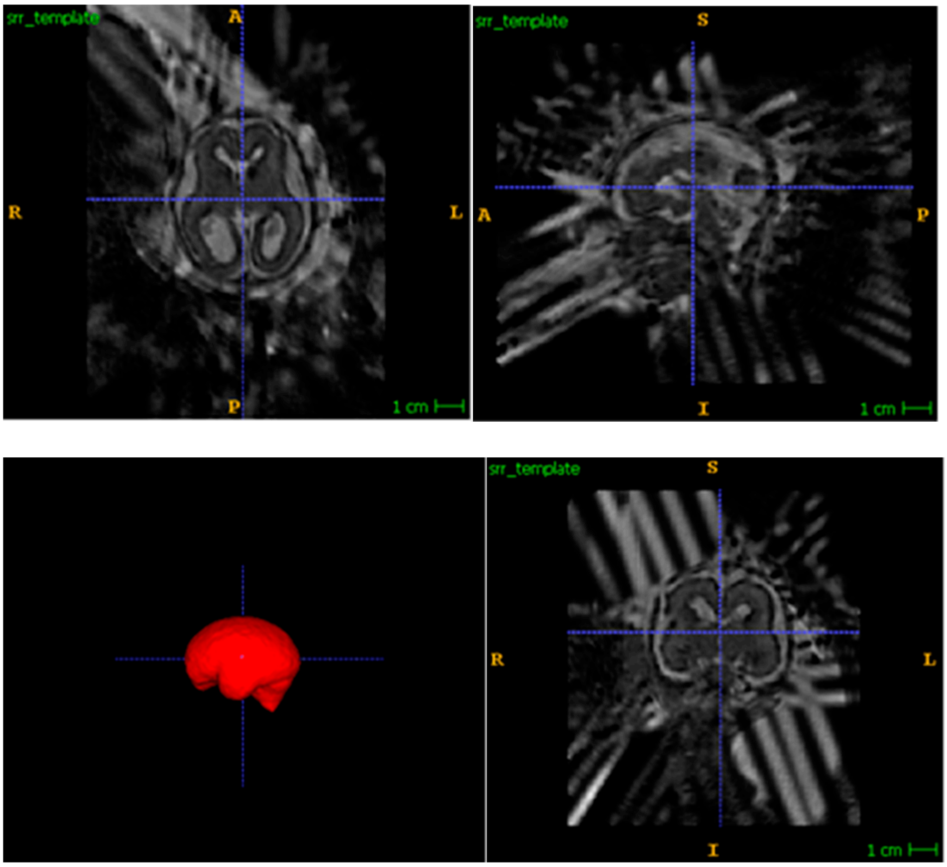

Supplement Figure S6 - Option 2 for selecting cuts for 3D reconstruction.

Option 3 for selecting cuts:

As an alternative approach, we explored the possibility of reconstructing the fetal brain MRI using only five axial images, without including the coronal or sagittal axes (Supplement Figure S7). Specifically, we reconstructed the 35/47/44/49/38 axial series, which took approximately 22 minutes. The results of this reconstruction are as follows:

- In terms of axial image quality, there was no significant improvement compared to the previous options for reconstruction.

- However, it is important to note that the quality of the coronal and sagittal views was neglected in this configuration. Since our primary focus was on measuring the Ventricle, which primarily relies on axial images, the compromised quality in the coronal and sagittal views was deemed acceptable for the specific analysis conducted in our study.

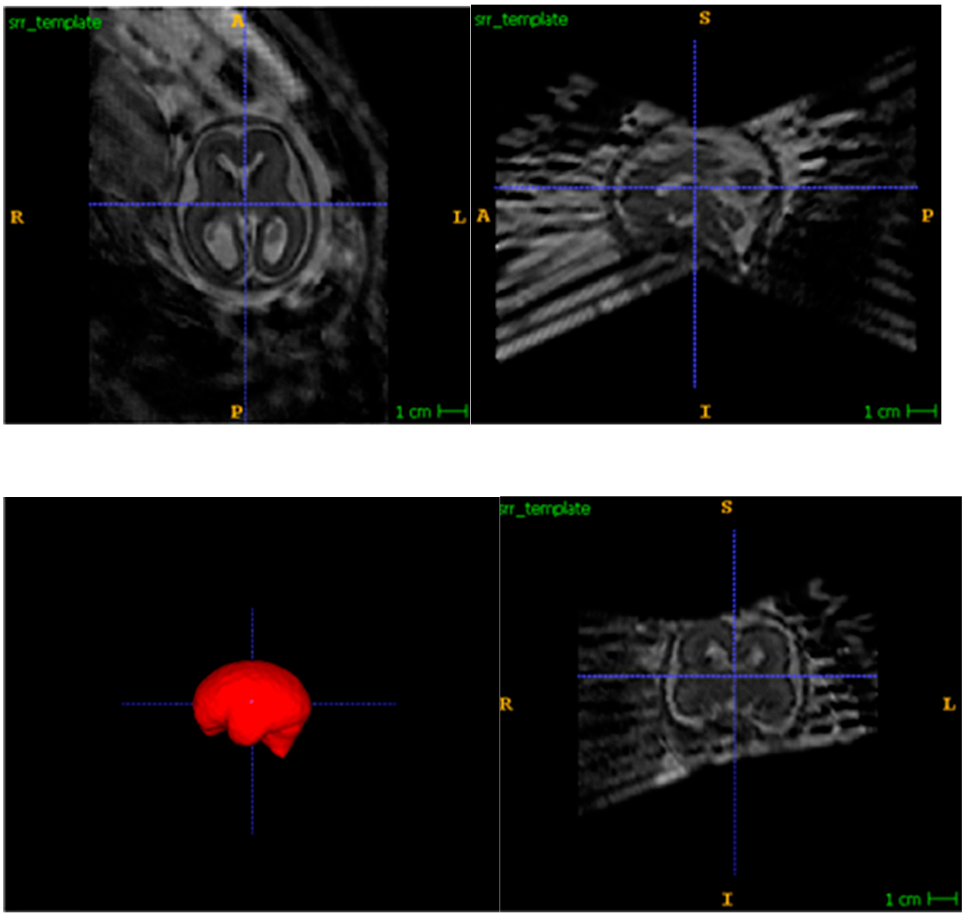

Supplement Figure S7 - Option 3 for selecting cuts for 3D reconstruction

- Finally, after considering different options, we ultimately chose option 2 for the reconstruction process. This decision was driven by the significant time savings of 20 minutes compared to using 9 stacks, while still maintaining almost the same quality in

the axial images. Given that our project focused on measuring the Ventricle, the slightly compromised quality of the sagittal and coronal images was not deemed critical.

- It is worth noting that the cuts selected by our AI model and those manually selected by radiologists and neuroradiologists were independently chosen, even from different sequences. However, there were notable similarities between the AI-selected cuts and the manual selections, particularly regarding the Ventricle's outline and the presence of dark areas within it (Supplement Figure S8) .These similarities provide further validation of the accuracy and effectiveness of our AI model in identifying relevant anatomical structures during the reconstruction process.

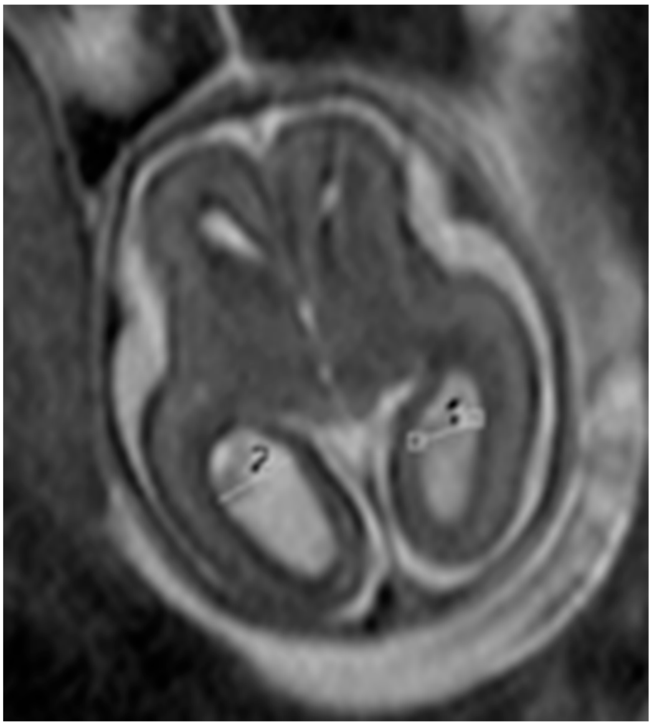

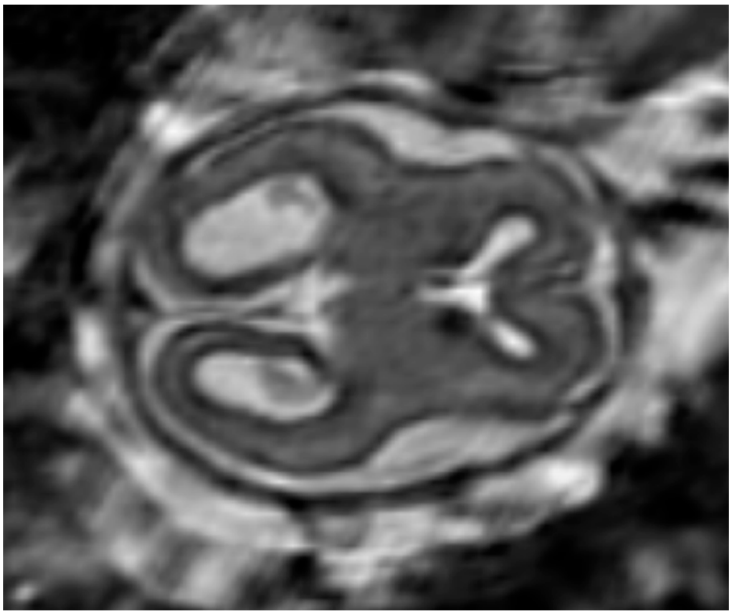

Supplement Figure S8- Upper: Manually selected label slice from series 49, instance 16

Below: AI-selected slices from five series of reconstructions, including axial 35, 46, 44, sagittal 34, and coronal 42.

The intensity and orientation of the image are automatically adjusted.
