# Supplementary material for: Automatic Ventriculomegaly Detection in Fetal Brain MRI: A Step-by-Step Deep Learning Model for Novel 2D-3D Linear Measurements
Source: Diagnostics (Basel). 2023 Jul 13;13(14):2355. doi: 10.3390/diagnostics13142355 (PMC10378043; doi:10.3390/diagnostics13142355)
Supplement: Supplementary file 1 [file diagnostics-13-02355-s001.zip › Source code.pdf]

In [1]:
```
# !pip install monai
```

In [2]:
```
import warnings

warnings.filterwarnings("ignore")

import os
from glob import glob

import matplotlib.pyplot as plt
import numpy as np
from monai.config import print_config
from sklearn.metrics import mean_squared_error

print_config()
```

```
MONAI version: 1.1.0
Numpy version: 1.23.5
Pytorch version: 1.13.1.post200
MONAI flags: HAS_EXT = False, USE_COMPILED = False, USE_META_DICT = False
MONAI rev id: a2ec3752f54bfc3b40e7952234fbeb5452ed63e3
MONAI __file__: /home/achilles/miniconda3/envs/colab/lib/python3.9/site-packages/mon
ai/__init__.py

Optional dependencies:
Pytorch Ignite version: 0.4.8
Nibabel version: 5.0.1
scikit-image version: 0.19.3
Pillow version: 9.4.0
Tensorboard version: 2.11.0
gdown version: NOT INSTALLED or UNKNOWN VERSION.
TorchVision version: 0.14.1a0+b69fce3
tqdm version: 4.65.0
lmdb version: NOT INSTALLED or UNKNOWN VERSION.
psutil version: 5.9.4
pandas version: 1.5.3
einops version: NOT INSTALLED or UNKNOWN VERSION.
transformers version: NOT INSTALLED or UNKNOWN VERSION.
mlflow version: NOT INSTALLED or UNKNOWN VERSION.
pynrrd version: NOT INSTALLED or UNKNOWN VERSION.

For details about installing the optional dependencies, please visit:
    https://docs.monai.io/en/latest/installation.html#installing-the-recommended-dep
endencies
```

In [3]:
```
import time
total_start = time.time()
```

In [4]:
```
# from google.colab import drive
# drive.mount('/content/gdrive')
# workpath = '/content/gdrive/MyDrive/Workspace'
# gData = '/content/gdrive/MyDrive/Workspace/DataSet'
# os.chdir('/content/gdrive/MyDrive/Workspace/JupyterLab/MONAI/fetal/v9')
```

In [5]:
```
curpath = os.getcwd()
workpath = curpath.split("Workspace")[0]
gData = os.path.join(workpath, "Workspace", "DataSet")
```

In [6]:
```
# test_dir = os.path.join(gData, "rush", "fetaldata", "20221011_xuchu_256_fold4")
# test_dir = os.path.join(gData, "rush", "fetaldata", "20230130_asher_256_fold4")
test_dir = os.path.join(gData, "rush", "fetaldata", "20230306_5_3ax_haste_256_fold4
model_dir = os.path.join(os.getcwd(), "brats")
```

In [7]:
```
path_test_raws = sorted(glob(test_dir + os.sep + "*_256.nii.gz"))
path_test_masks = sorted(glob(test_dir + os.sep + "*_seg_7.nii.gz"))

test_files = [
    {"vol": raw_name, "mask": mask_name}
    for raw_name, mask_name in zip(path_test_raws, path_test_masks)
]
```

In [8]:
```
# test_files = test_files[0:1]
```

In [9]:
```
from monai.inferers import sliding_window_inference
from monai.transforms import (
    Activations,
    AsDiscrete,
    Compose,
    EnsureChannelFirstd,
    Invertd,
    KeepLargestConnectedComponent,
    LoadImaged,
    MapTransform,
    NormalizeIntensityd,
    Orientationd,
    ResizeWithPadOrCropd,
    Spacingd,
    ToTensord,
)
```

In [10]:
```
KEYS = ("vol", "mask")

test_transforms = Compose(
    [
        LoadImaged(KEYS),
        EnsureChannelFirstd(KEYS),
        Orientationd(KEYS, axcodes="LAS"),
        NormalizeIntensityd(keys=["vol"], nonzero=True, channel_wise=True),
        ToTensord(KEYS),
    ]
)
```

In [11]:
```
test_dict = test_transforms(test_files)
```

In [12]:
```
index = 0
each_test = test_dict[index]
print(index, each_test["vol"].meta["filename_or_obj"])
plt.figure("check", (12, 12))
plt.subplot(2, 2, 1)
plt.imshow(each_test['vol'][0, ..., 128], cmap="gray")
plt.title("AXIAL")
plt.subplot(2, 2, 2)
plt.imshow(each_test['vol'][0, 128, ...], cmap="gray")
plt.title("SAGITTAL")
plt.subplot(2, 2, 4)
plt.imshow(each_test['vol'][0, :, 128, :], cmap="gray")
plt.title("CORONAL")
plt.show()
```

```
0 /mnt/Storage/Xuchu_Liu/Workspace/DataSet/rush/fetaldata/20230306_5_3ax_haste_256_f
old4/8187391_256.nii.gz
```

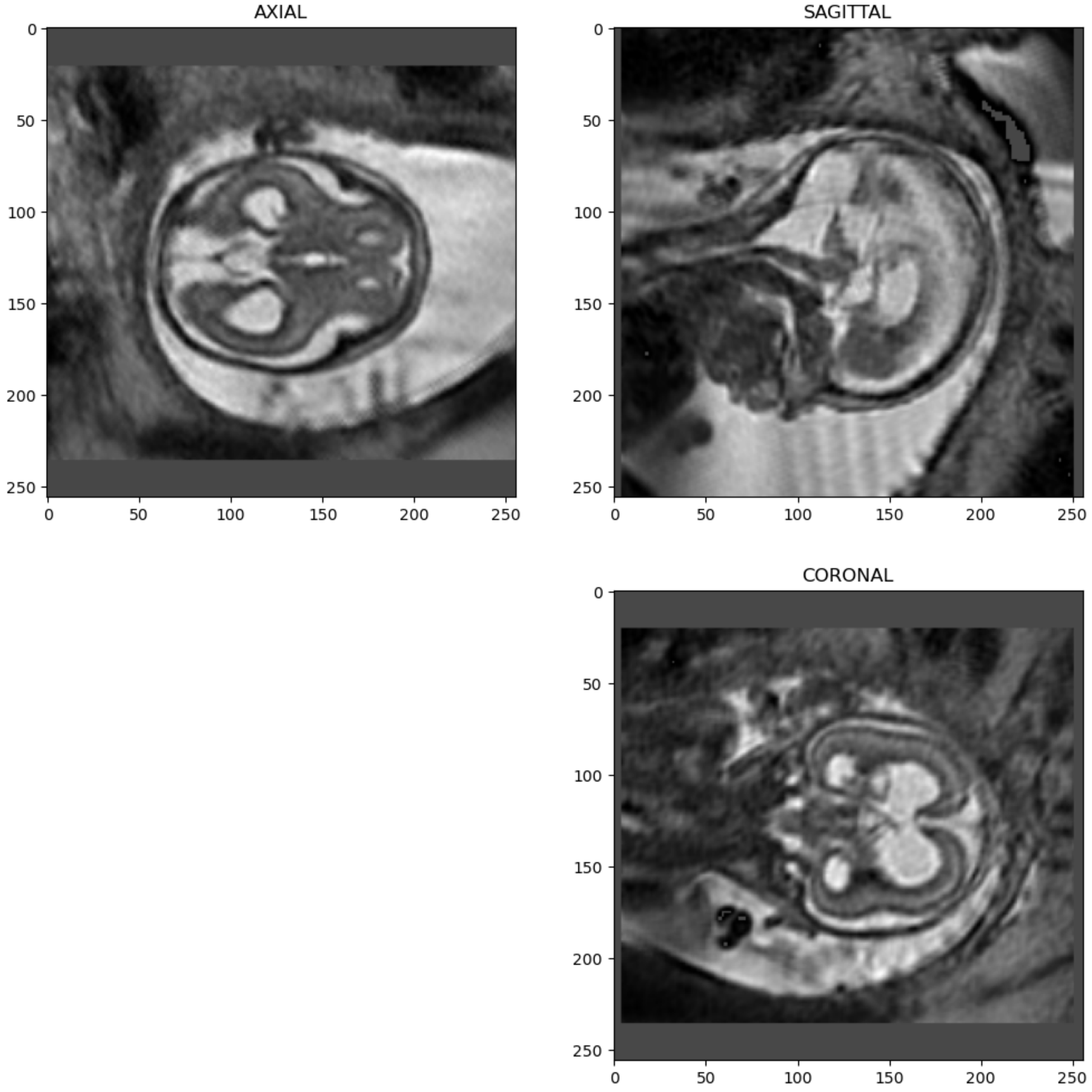

In [13]:
```
import cv2
import matplotlib.patches as mpatches
import matplotlib.patches as patches
import torch
from matplotlib.colors import ListedColormap
```

In [14]:
```
def horizontalsymmetry(data):
    Y, X = data.shape
    c_Y = Y // 2
    loss = 0
    up = data[0:c_Y, :150]
    down = data[c_Y:, :150]
    diff = up ^ torch.flip(down, dims=[0])
    loss = torch.count_nonzero(diff)
    return loss
```

In [15]:
```
quantile_value = 0.3
mean_ratio = 0.9
col_sum_max_ratio = 0.9
def ven_measurement(data):
    # get the bright ventricle part
    i_data = data.ravel()[np.flatnonzero(data)]
    # a = np.quantile(i_data, quantile_value)
    a = np.mean(i_data) * mean_ratio
    v_data = np.where(data < a, 0, 1.0)
    v_data_0 = v_data[0]

    # find the largest white part
    v_env_0 = np.zeros_like(v_data_0)
    v_mix_0 = np.zeros_like(v_data_0)
    cv_data = np.transpose(v_data, (1, 2, 0))
    contours, hierarchy = cv2.findContours(cv_data.astype("uint8"), cv2.RETR_TREE,
    area = []
    for k in range(len(contours)):
        area.append(cv2.contourArea(contours[k]))
    max_idx = np.argmax(np.array(area))
    cv2.drawContours(v_env_0, contours, max_idx, 1, cv2.FILLED)
    del area
    v_mix_0 = v_data_0 * v_env_0

    cv_rect = cv2.minAreaRect(contours[max_idx])
    [c_X, c_Y], [H, W], A = cv_rect
    if A > 45:
        A_cur = A - 90
    else:
        A_cur = -A
    if H > W:
        A_cur = -A_cur
    # print(cv_rect, A_cur)
    M_cur = cv2.getRotationMatrix2D(cv_rect[0], A_cur, 1.0)
    rotated_env_0 = cv2.warpAffine(v_env_0, M_cur, (256, 256))
    rotated_env_1 = np.where(rotated_env_0 > 0, 1, 0) # convert to [0, 1]
    rotated_mix_0 = cv2.warpAffine(v_mix_0, M_cur, (256, 256))
    rotated_mix_1 = np.where(rotated_mix_0 > 0, 1, 0) # convert to [0, 1]
```

```
    col_sum = np.sum(rotated_env_1, axis=0)
    col_sum_max = np.max(col_sum)
    col_sum_max_idx = np.where(col_sum == col_sum_max)
    col_sum_mix = np.sum(rotated_mix_1, axis=0)
    col_sum_threshold = np.floor(col_sum_max * col_sum_max_ratio)
    for col_sum_ven_idx in range(col_sum_max_idx[0][0], 130):
        if col_sum_mix[col_sum_ven_idx] < col_sum_threshold:
            break
    col_sum_ven_idx -= 1

    rotated_x = col_sum_ven_idx
    rotated_y = np.nonzero(rotated_mix_1[:, rotated_x])
    rotated_y_up = rotated_y[0][0]
    rotated_y_down = rotated_y[0][-1]
    ventricle_len = rotated_y_down - rotated_y_up + 1

    W_up = rotated_x - c_X
    H_up = rotated_y_up - c_Y
    R_up = np.sqrt(W_up*W_up + H_up*H_up)
    alpha = np.arctan(H_up/W_up)
    theta = A_cur * np.pi/180
    gamma = alpha + theta
    correct_x_up = int(c_X + R_up*np.cos(gamma))
    correct_y_up = int(c_Y + R_up*np.sin(gamma))

    W_down = rotated_x - c_X
    H_down = rotated_y_down - c_Y
    R_down = np.sqrt(W_down*W_down + H_down*H_down)
    alpha = np.arctan(H_down/W_down)
    theta = A_cur * np.pi/180
    gamma = alpha + theta
    correct_x_down = int(c_X + R_down*np.cos(gamma))
    correct_y_down = int(c_Y + R_down*np.sin(gamma))

    rotated_mix_1[:, rotated_x] = 0
    return rotated_mix_1, ventricle_len, correct_x_up, correct_y_up, correct_x_down
```

In [16]:

```
index = 0
for each_test in test_dict:
    print(index, os.path.basename(each_test["vol"].meta["filename_or_obj"]))
    index += 1

    ecsf_mask = torch.where(each_test["mask"] == 1, 1, 0)
    gm_mask = torch.where(each_test["mask"] == 2, 1, 0)
    wm_mask = torch.where(each_test["mask"] == 3, 1, 0)
    ventricles_mask = torch.where(each_test["mask"] == 4, 1, 0)
    cerebellum_mask = torch.where(each_test["mask"] == 5, 1, 0)
    deep_gm_mask = torch.where(each_test["mask"] == 6, 1, 0)
    brainstem_mask = torch.where(each_test["mask"] == 7, 1, 0)

    # find suitable slice index for measurement
    deep_gm_count = torch.count_nonzero(deep_gm_mask[0], dim=[0, 1])
    top_deep_gm_idx = torch.argsort(deep_gm_count, descending=True)
    best_deep_gm_idx = top_deep_gm_idx[0]
    min_s = 256 * 256
```

```
    for idx in range(best_deep_gm_idx, best_deep_gm_idx+1):

        # get largest_up and largest_down
        test_vol = each_test["vol"][..., idx]
        test_out = ventricles_mask[..., idx]
        largest_up = np.zeros((1, 256, 256))
        left_up = test_out[:, :127, :150]
        largest_left_up = KeepLargestConnectedComponent(applied_labels=1, connectiv
            left_up
        )
        largest_up[0, :127, :150] = largest_left_up
        largest_up = largest_up * each_test["vol"][..., idx]
        largest_up_0, up_ven_len, up_x1, up_y1, up_x2, up_y2 = ven_measurement(larg

        largest_down = np.zeros((1, 256, 256))
        left_down = test_out[:, 129:, :150]
        largest_left_down = KeepLargestConnectedComponent(applied_labels=1, connect
            left_down
        )
        largest_down[0, 129:, :150] = largest_left_down
        largest_down = largest_down * each_test["vol"][..., idx]
        largest_down_0, down_ven_len, down_x1, down_y1, down_x2, down_y2 = ven_meas

        print(f"{up_ven_len*0.5} {down_ven_len*0.5} {idx}")
        # print(f"slice: {idx} left: {down_ven_len*0.5} right: {up_ven_len*0.5}")
        plt.figure("check", (12, 12))
        plt.subplot(1, 1, 1)
        plt.imshow(each_test["vol"][0, ..., idx], cmap="gray")
        plt.plot([up_x1, up_x2], [up_y1, up_y2], color="red", linewidth=1)
        plt.text(up_x1+10, (up_y1+up_y2)//2, f"{up_ven_len*0.5}", color="w", fontsi
        plt.plot([down_x1, down_x2], [down_y1, down_y2], color="red", linewidth=1)
        plt.text(down_x2+10, (down_y1+down_y2)//2, f"{down_ven_len*0.5}", color="w"
        plt.title(f"AXIAL {idx}")
        # plt.subplot(1, 2, 2)
        # plt.imshow(ventricles_mask[0, ..., idx], cmap="gray")
        # plt.title(f"Ventricles")
        # ax = plt.subplot(1, 3, 3)
        # ax.imshow(largest_up_0 + largest_down_0, cmap="gray")
        plt.show()
    print("\n\n")
```

```
0 8187391_256.nii.gz
13.0 13.0 138
```

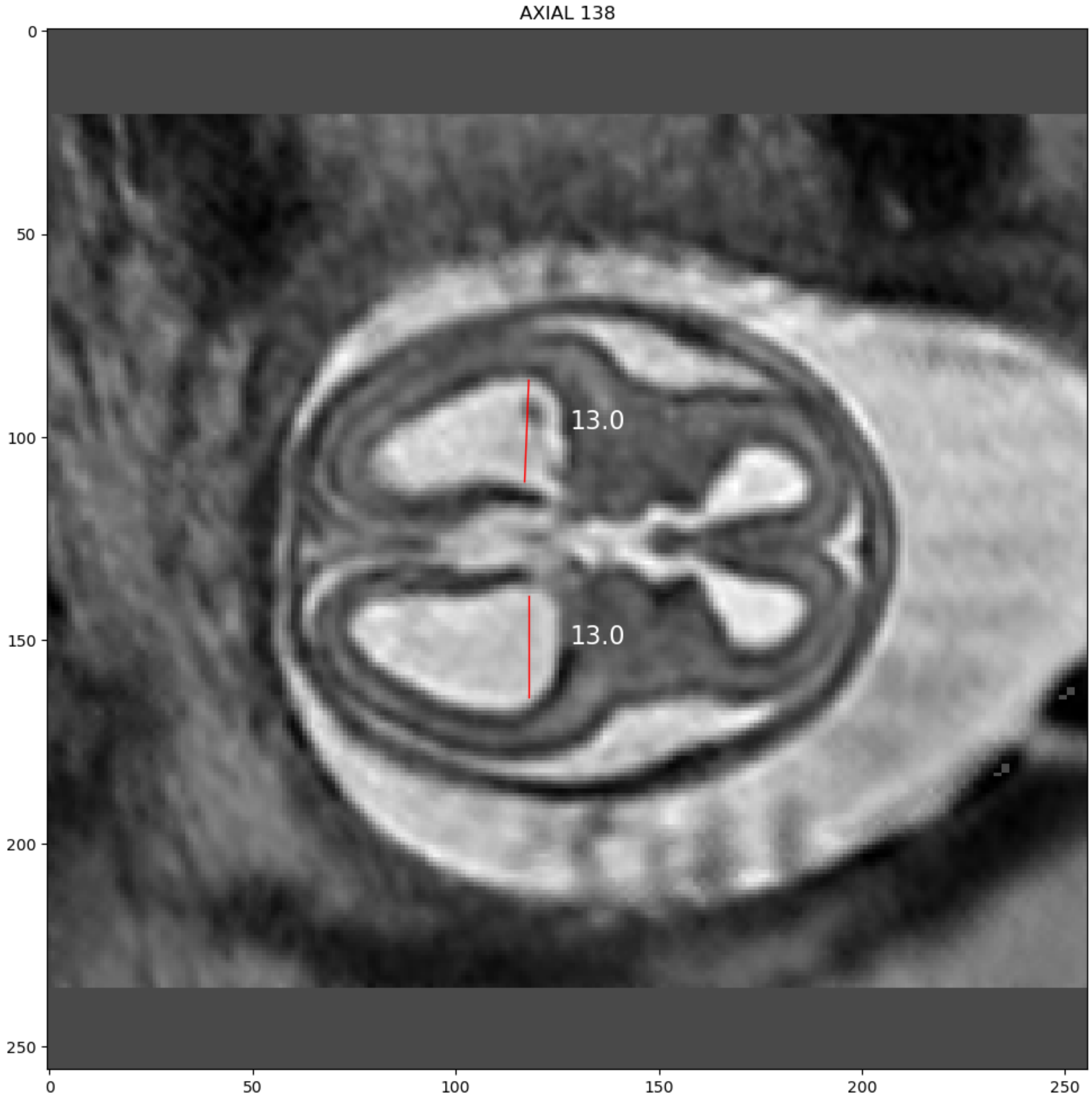


```
1 8275848_256.nii.gz
14.5 15.0 137
```

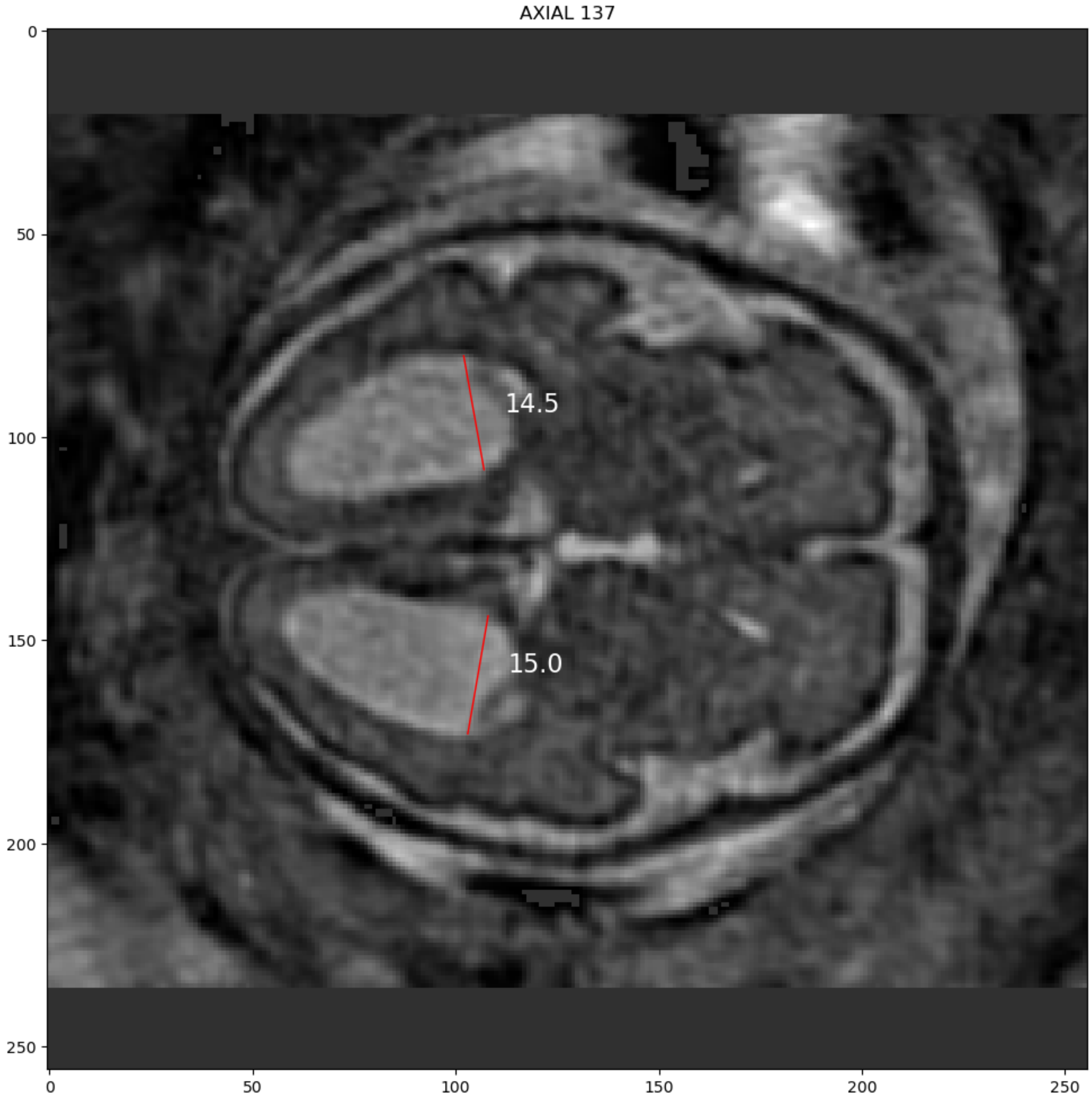


```
2 8330790_256.nii.gz
27.5 22.5 133
```

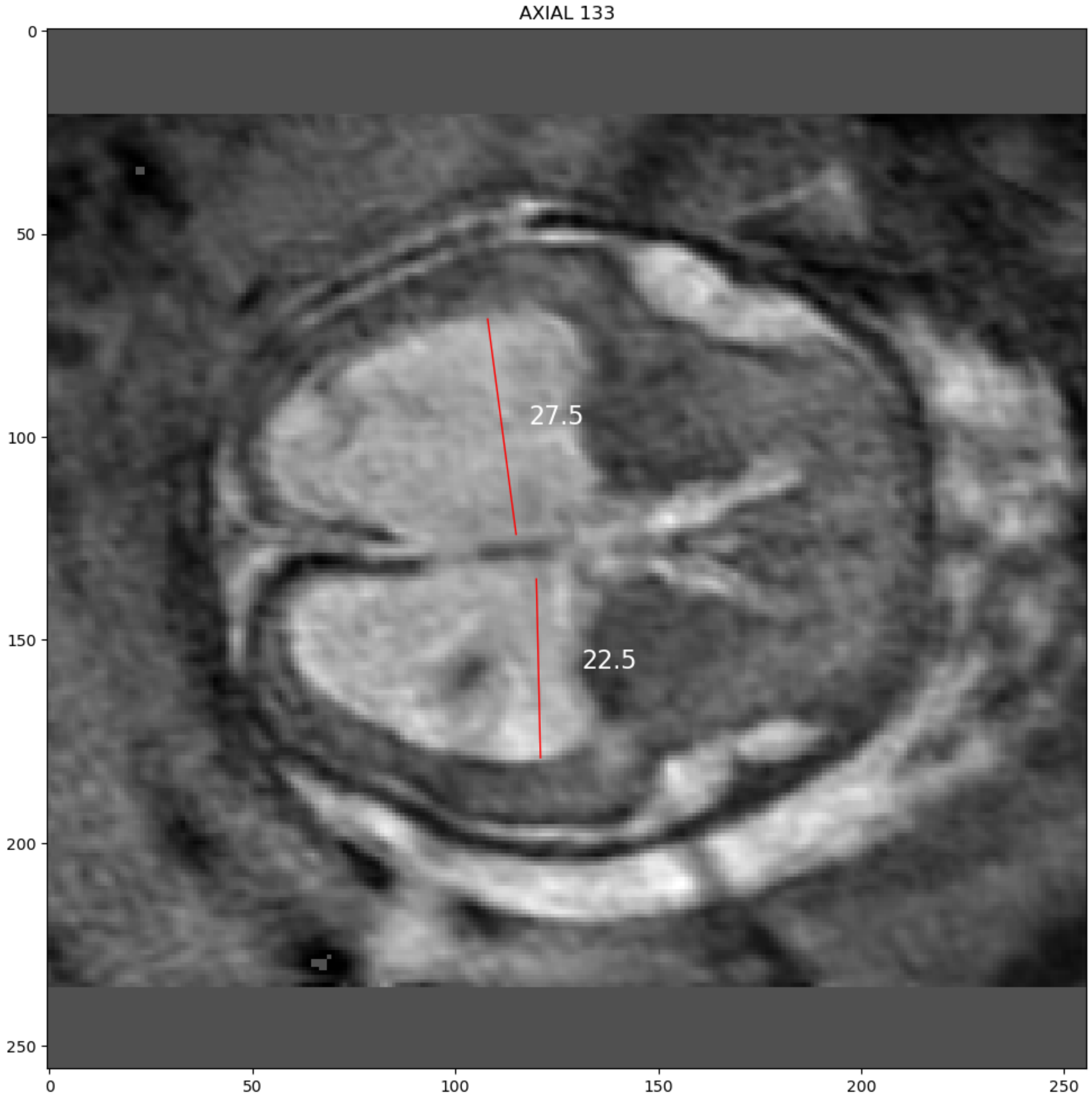


```
3 8360451_256.nii.gz
17.5 17.5 137
```

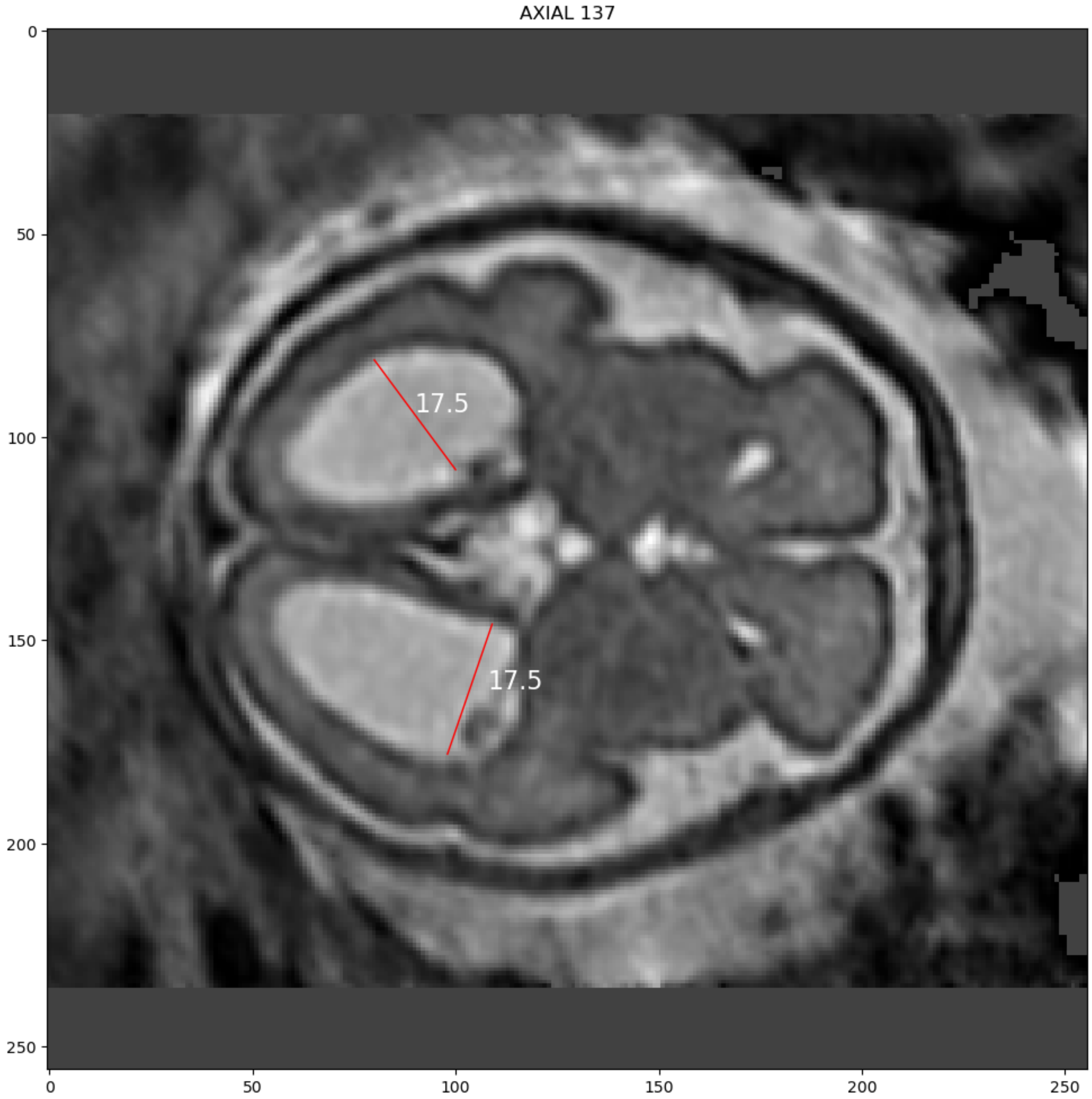


```
4 exam_000001_256.nii.gz
10.0 6.5 146
```

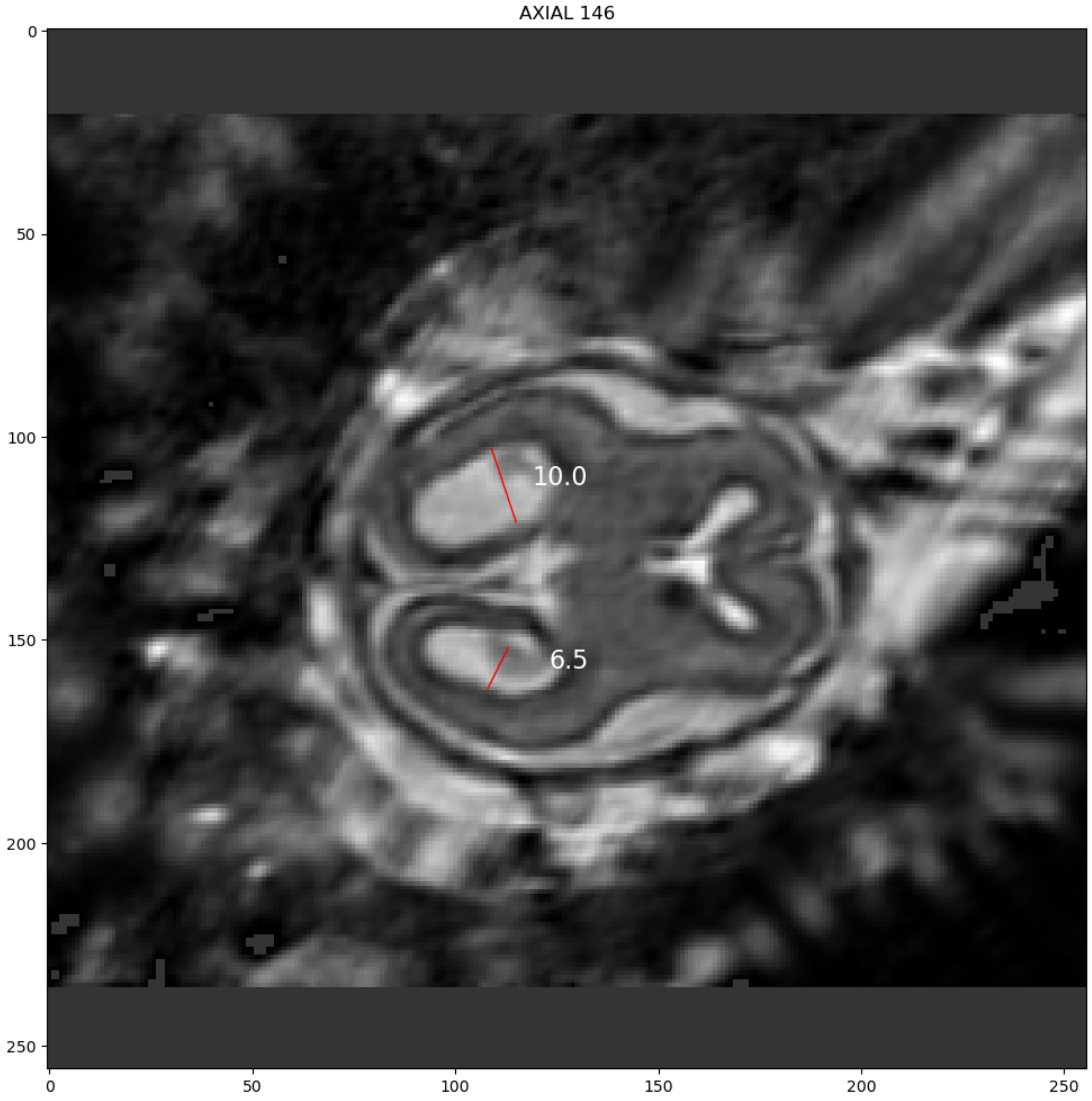


```
5 exam_000004_256.nii.gz
9.0 7.5 145
```

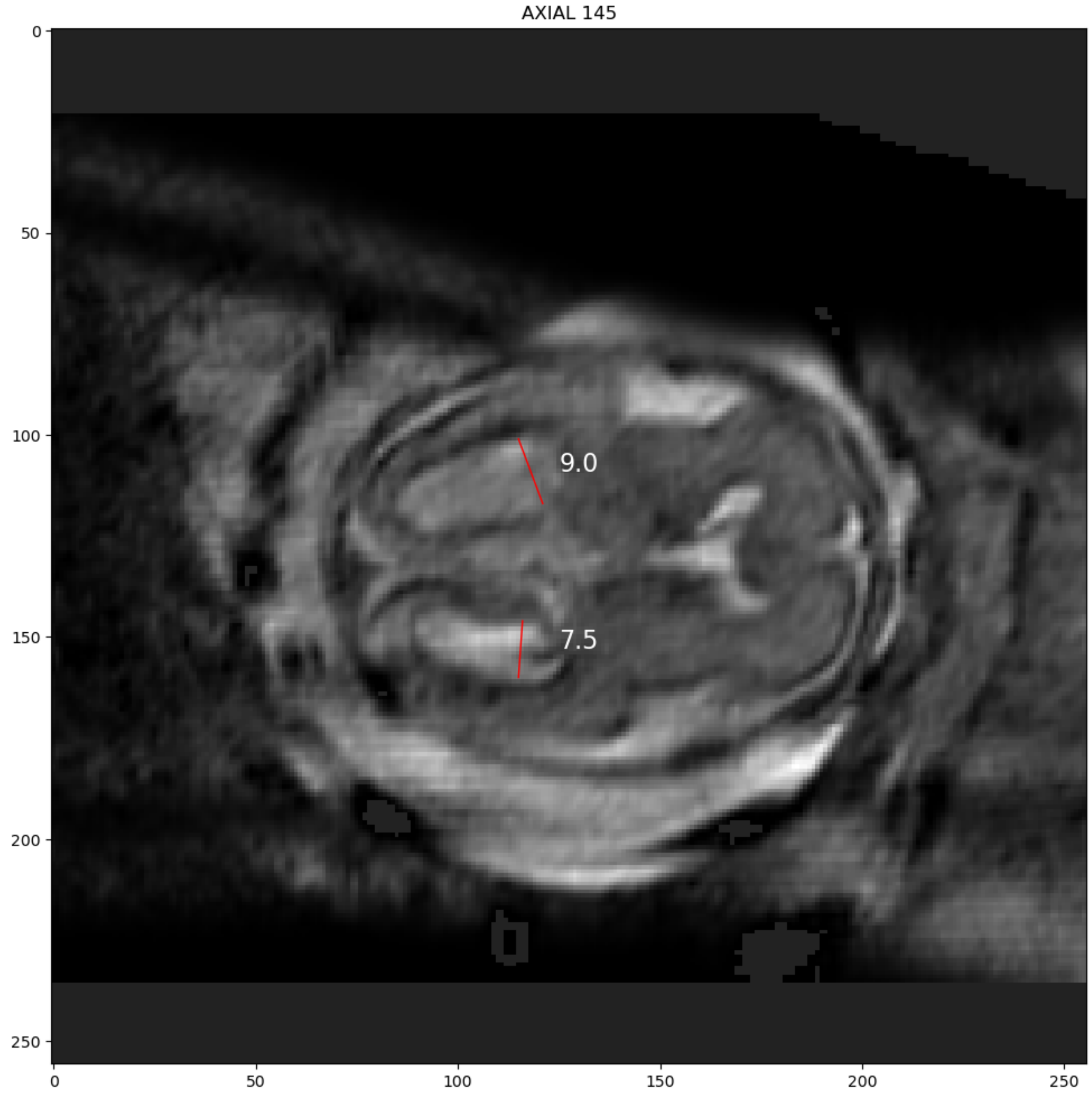


```
6 exam_000011_256.nii.gz
6.5 6.5 146
```

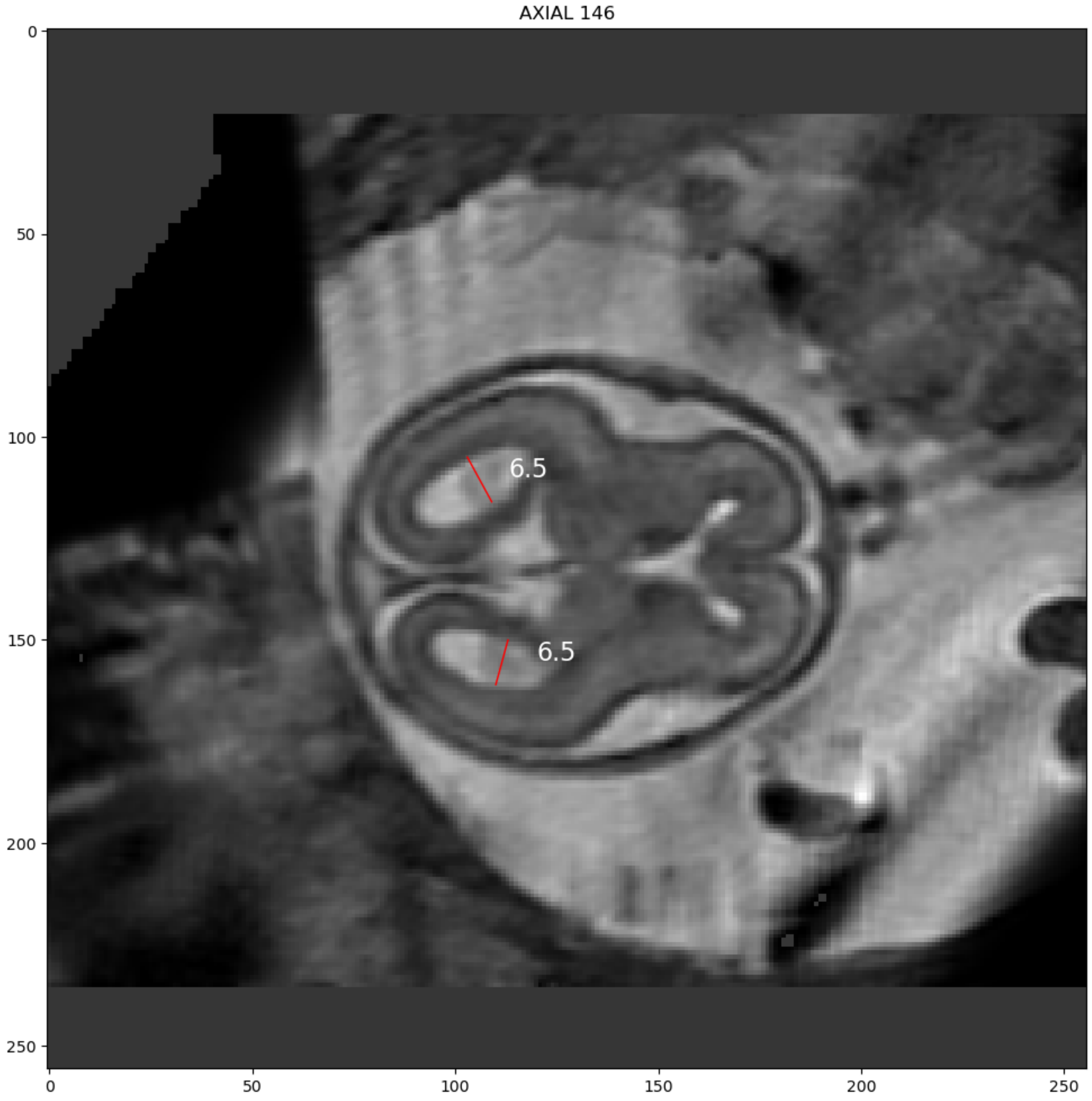


```
7 exam_000015_256.nii.gz
7.5 9.5 146
```

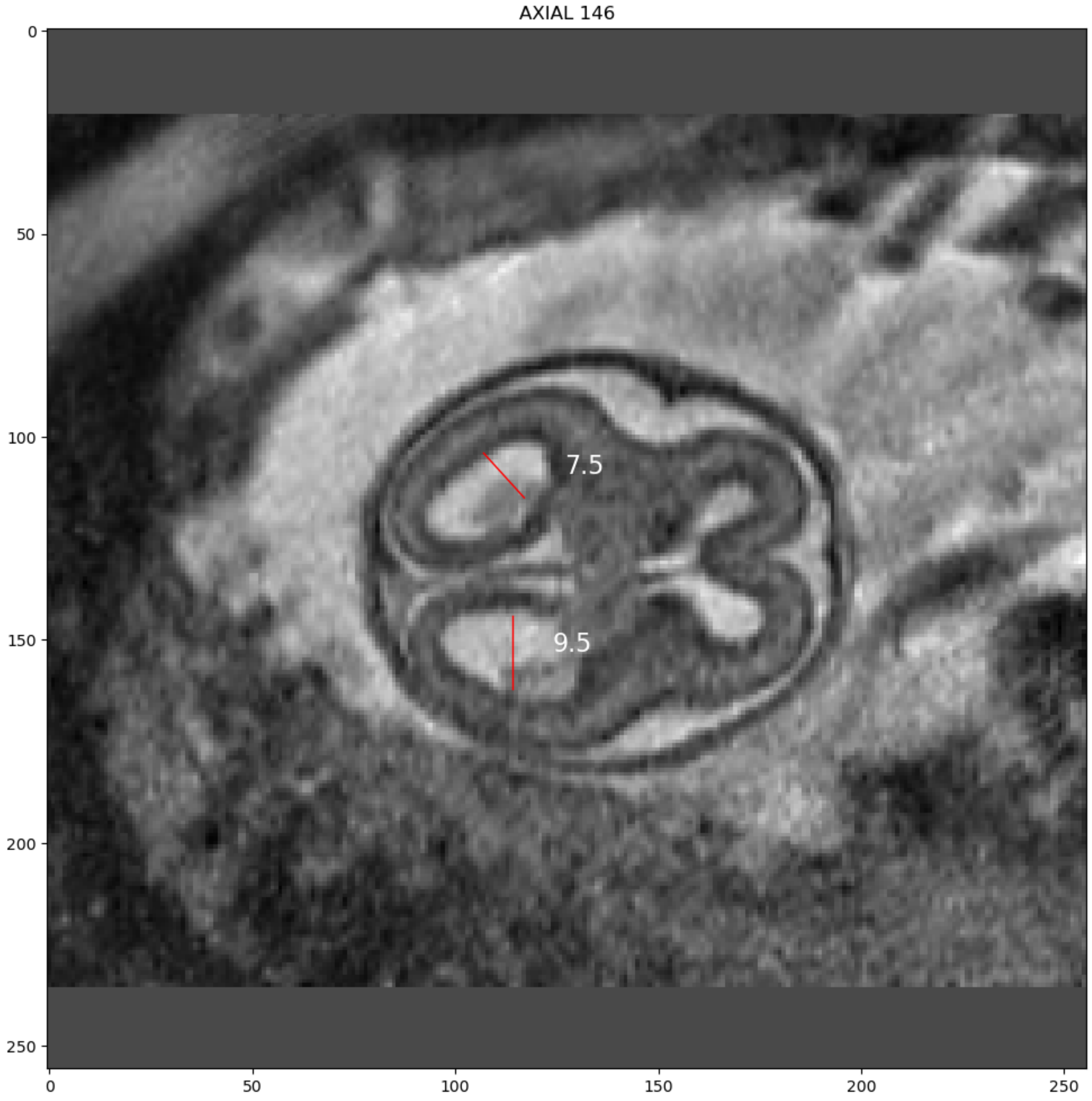


```
8 exam_000016_256.nii.gz
7.0 6.5 143
```

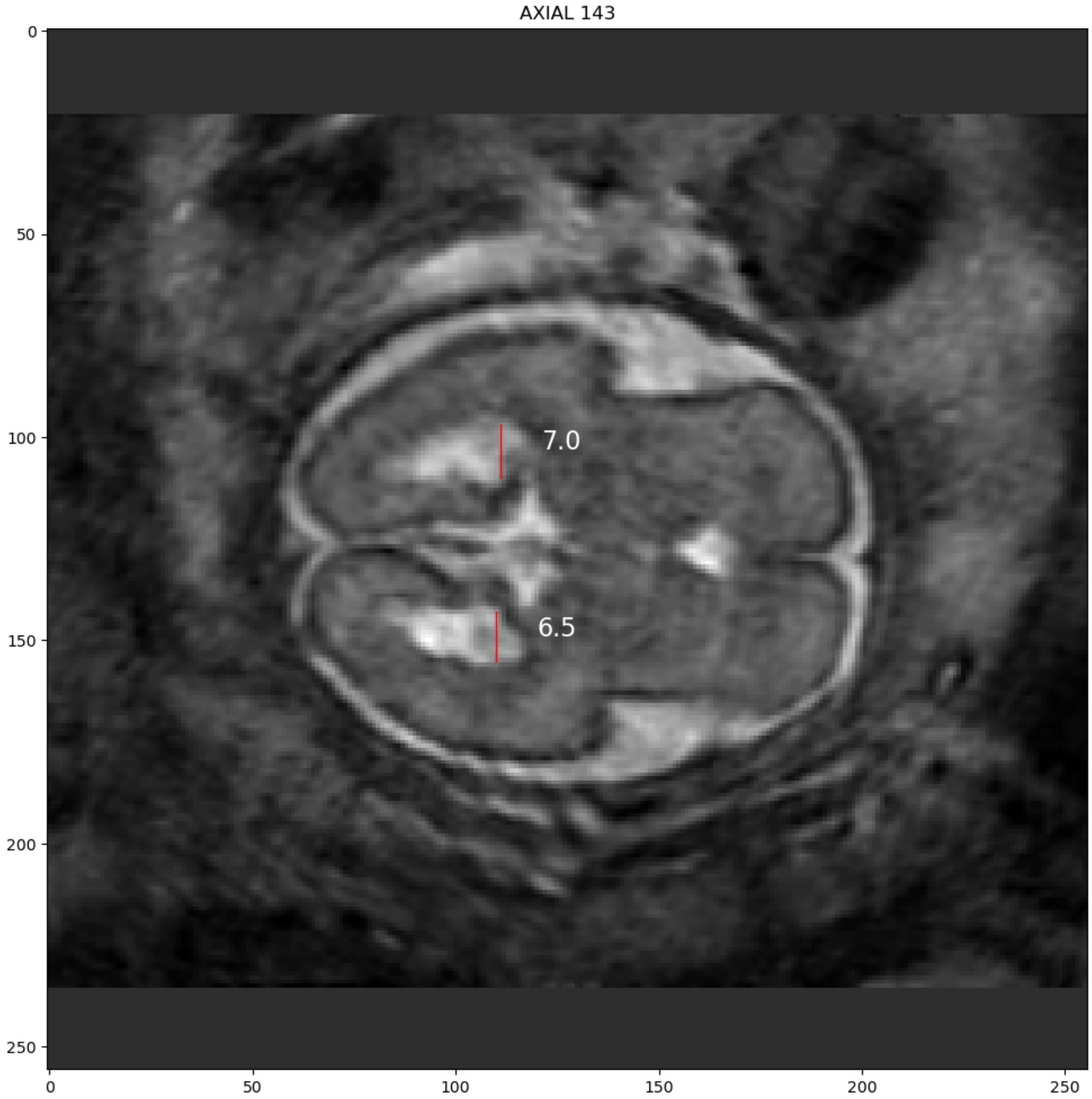


```
9 exam_000021_256.nii.gz
11.0 10.5 145
```

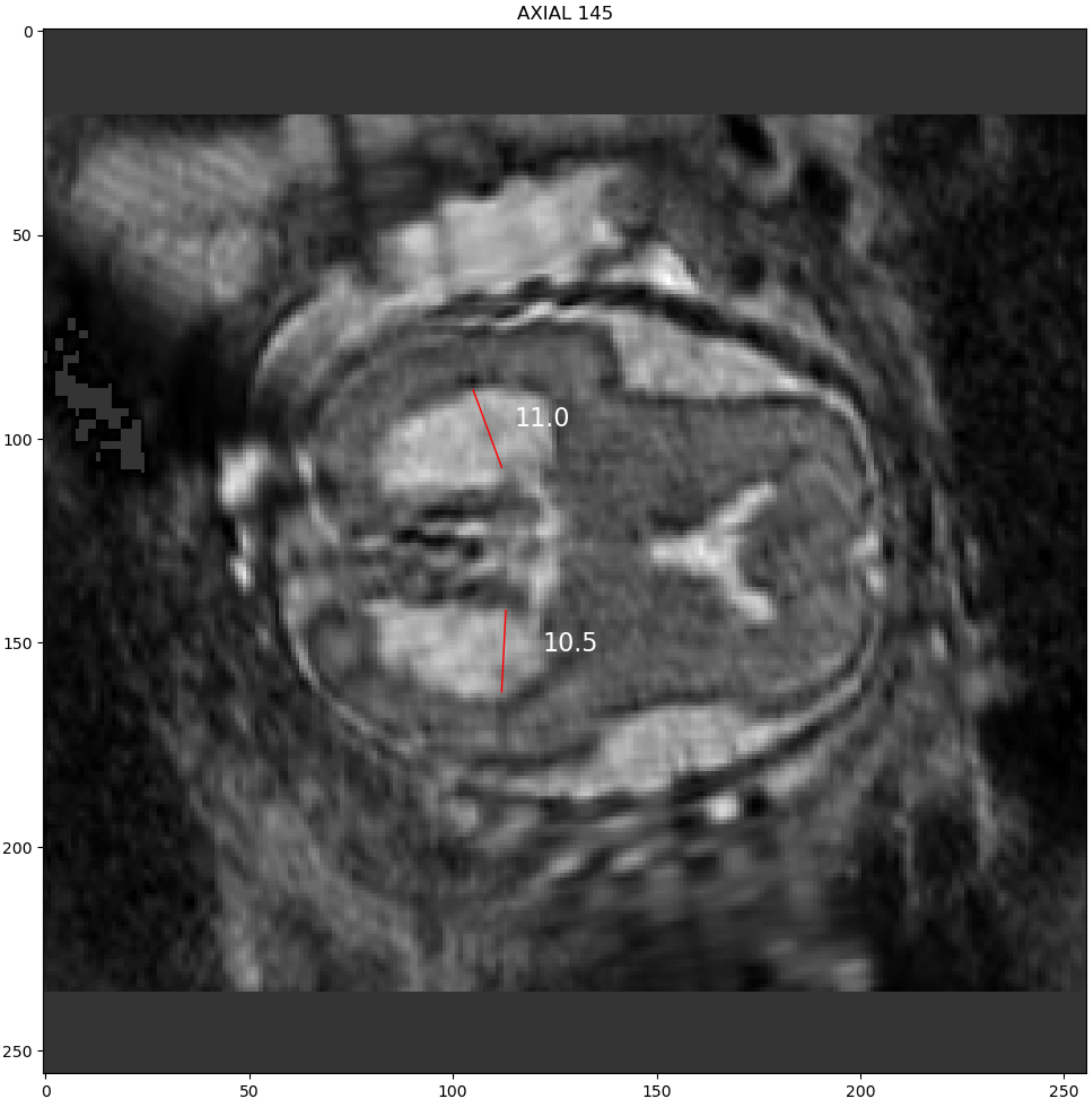


```
10 exam_000023_256.nii.gz
6.0 9.0 145
```

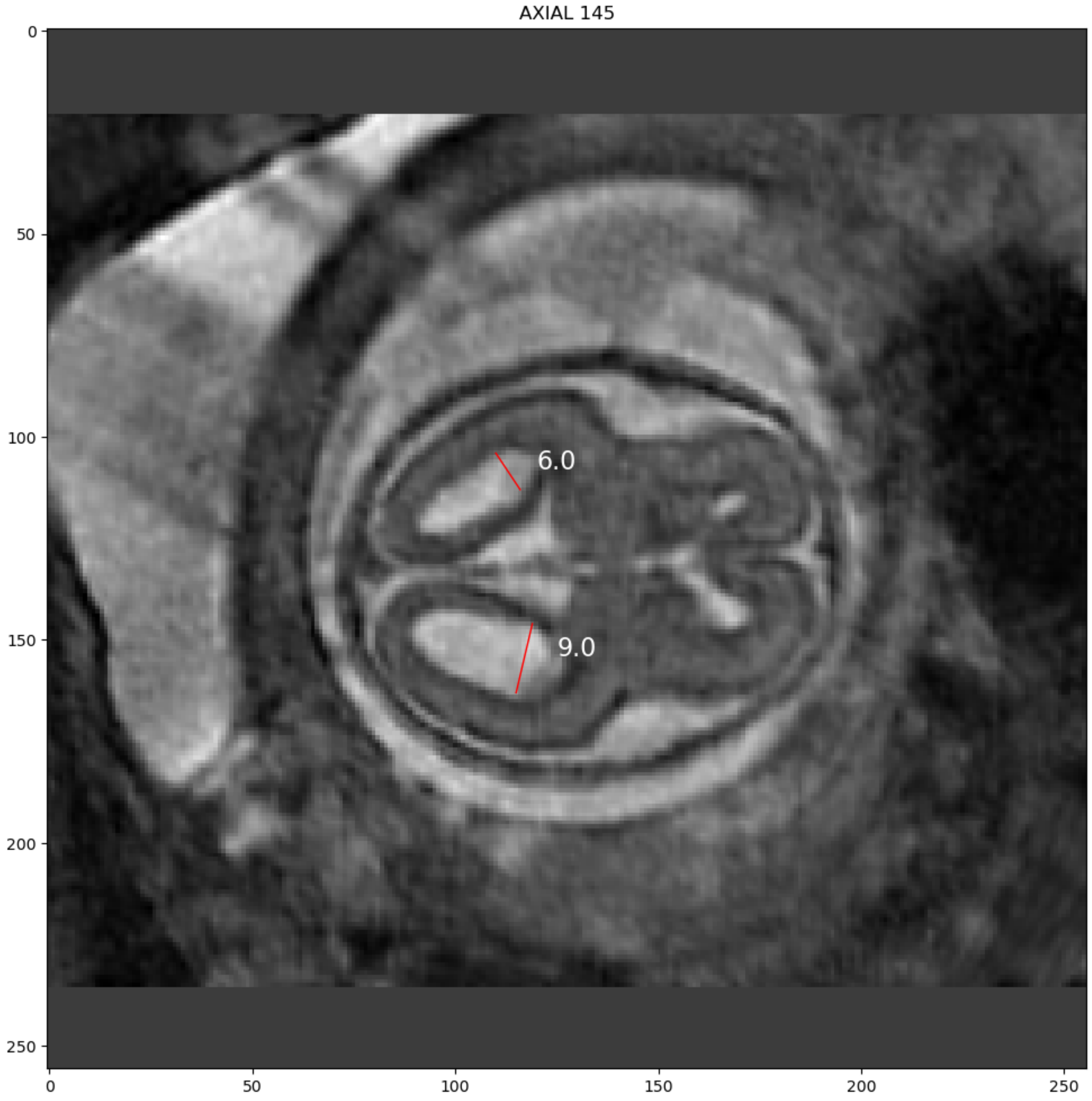


```
11 exam_000031_256.nii.gz
7.0 4.5 144
```

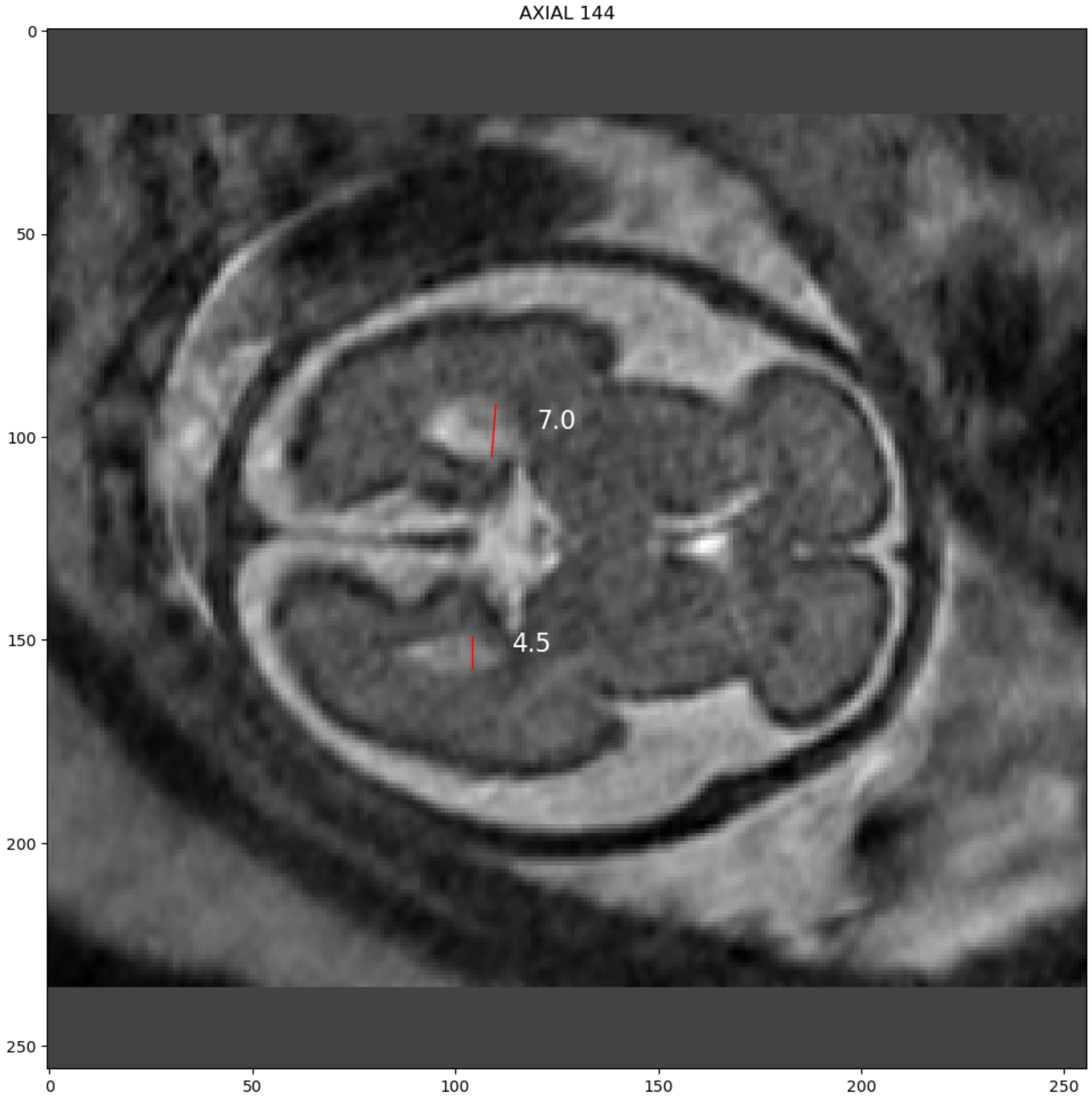


```
12 exam_000032_256.nii.gz
9.0 9.0 143
```

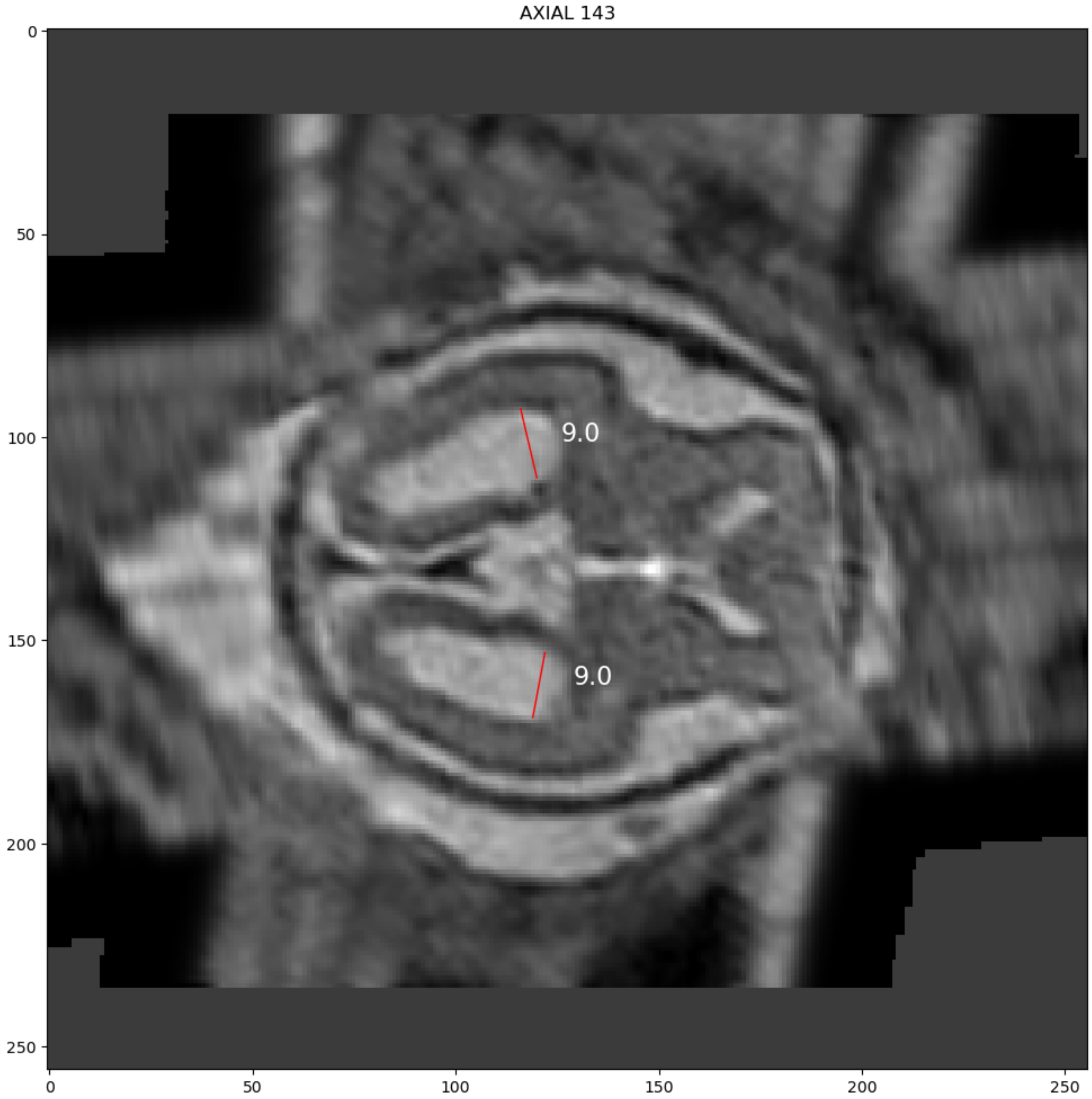


```
13 exam_000033_256.nii.gz
6.5 6.5 144
```

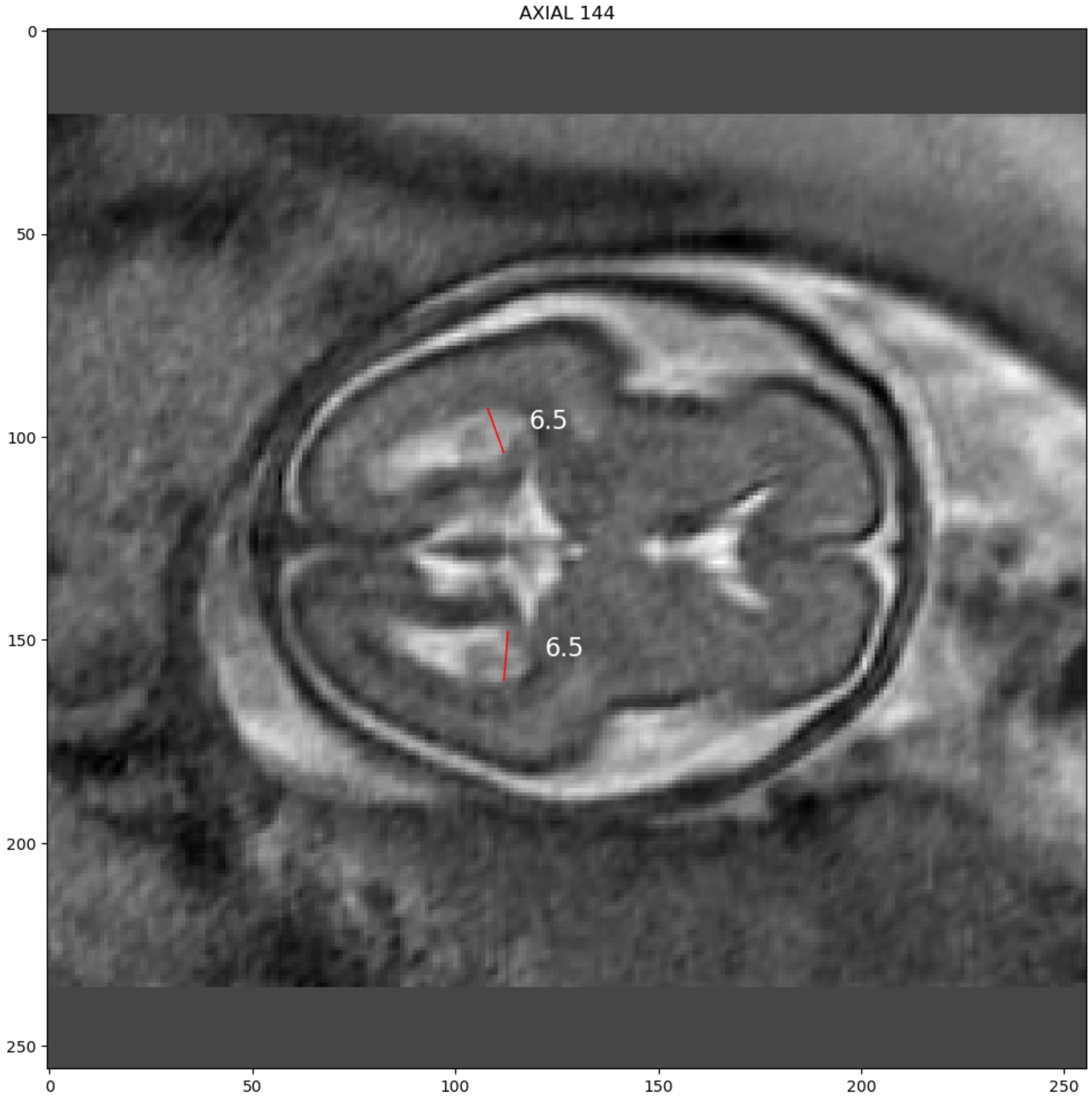


```
14 exam_000036_256.nii.gz
8.0 7.5 145
```

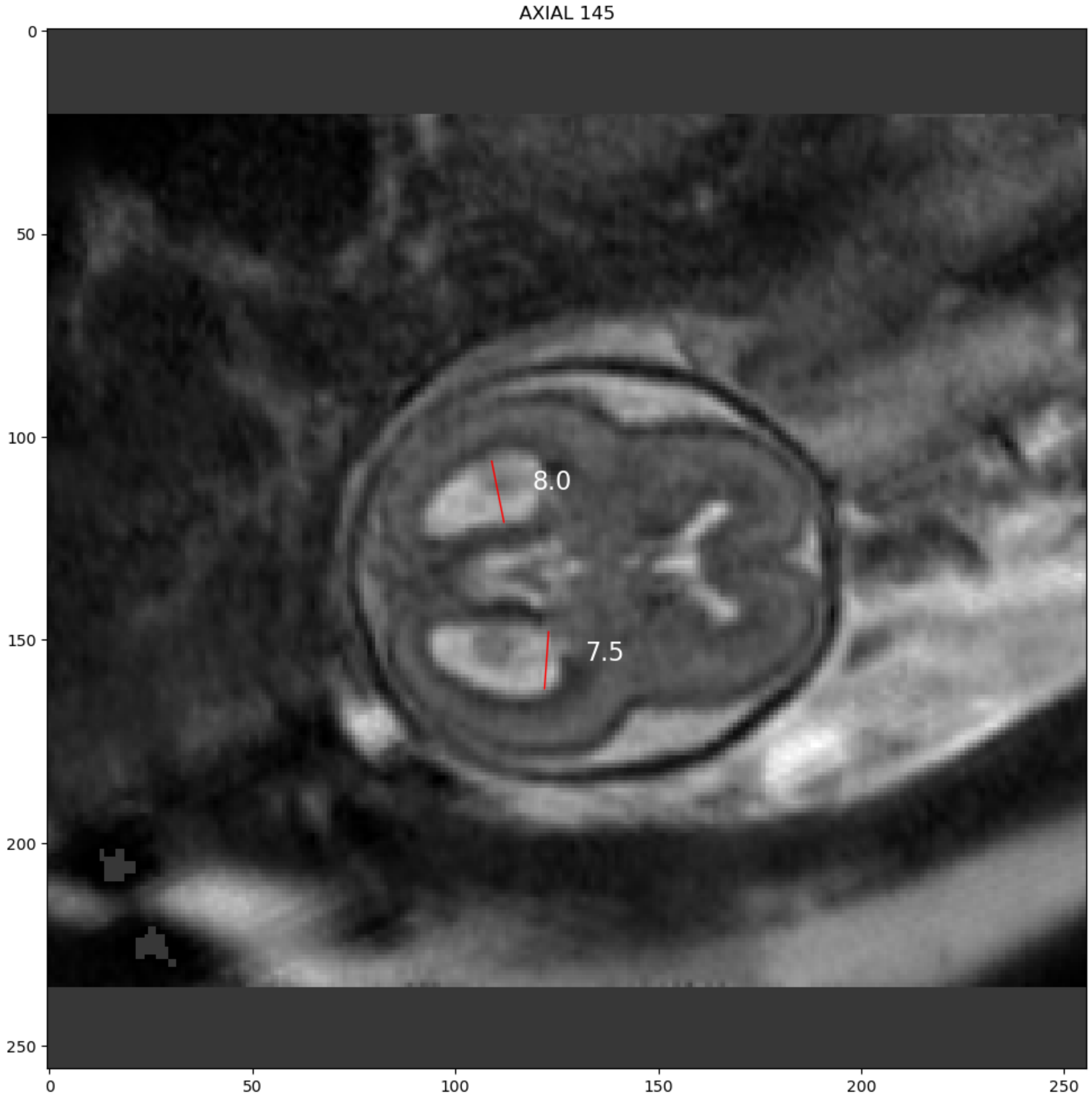


```
15 exam_000062_256.nii.gz
5.0 11.5 143
```

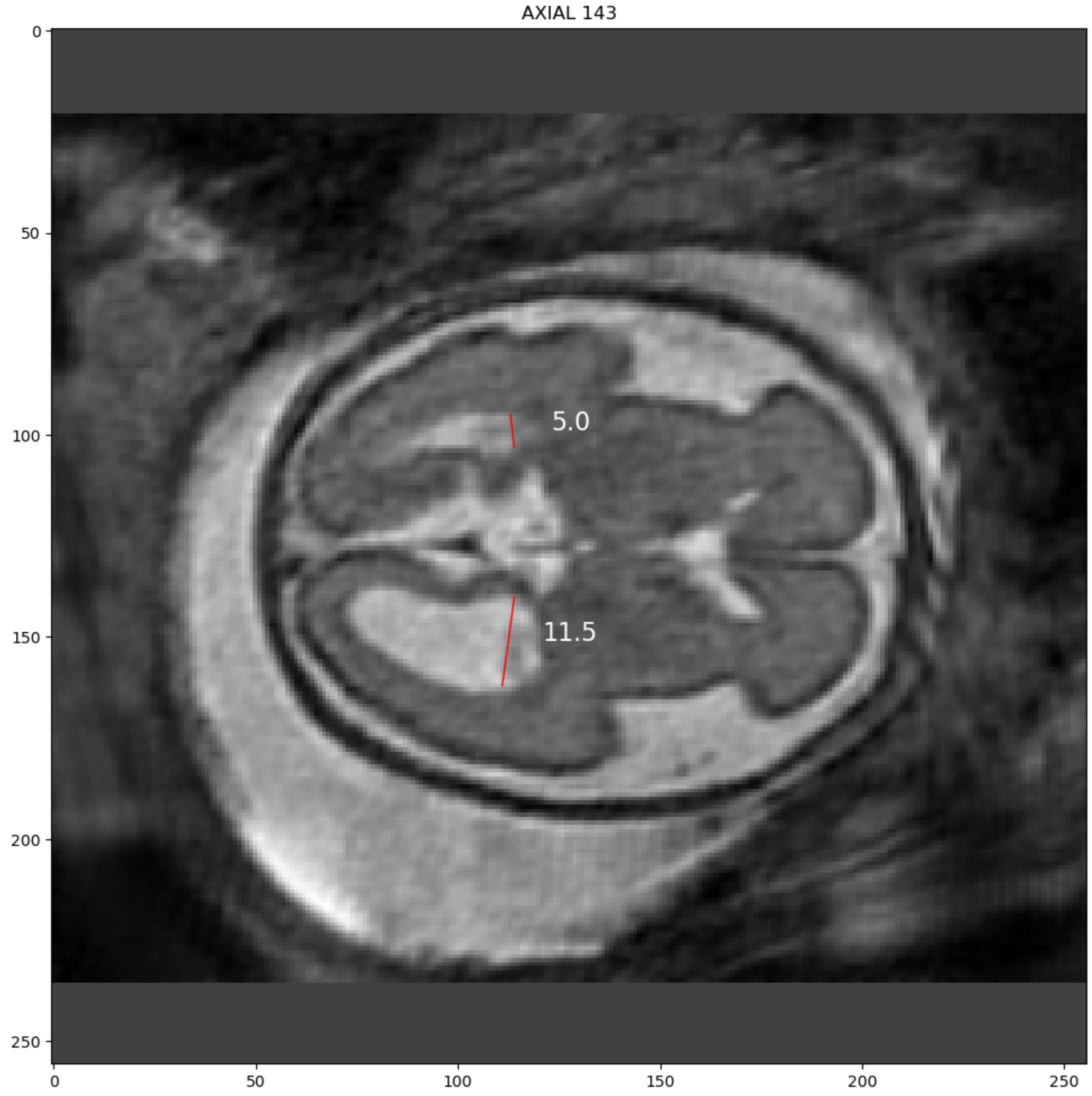


```
16 exam_000090_256.nii.gz
13.5 12.0 141
```

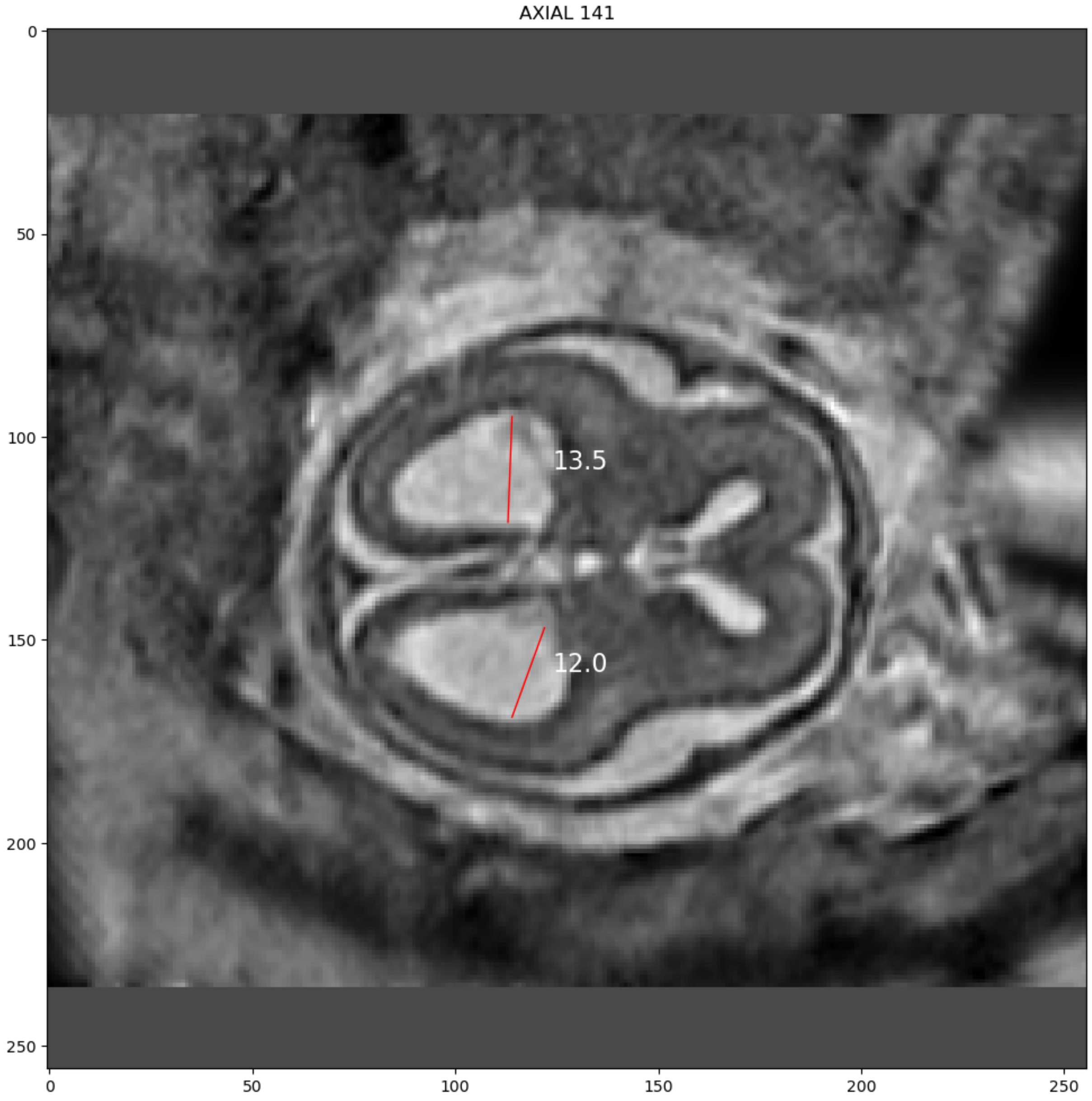


```
17 exam_000095_256.nii.gz
9.5 10.5 145
```

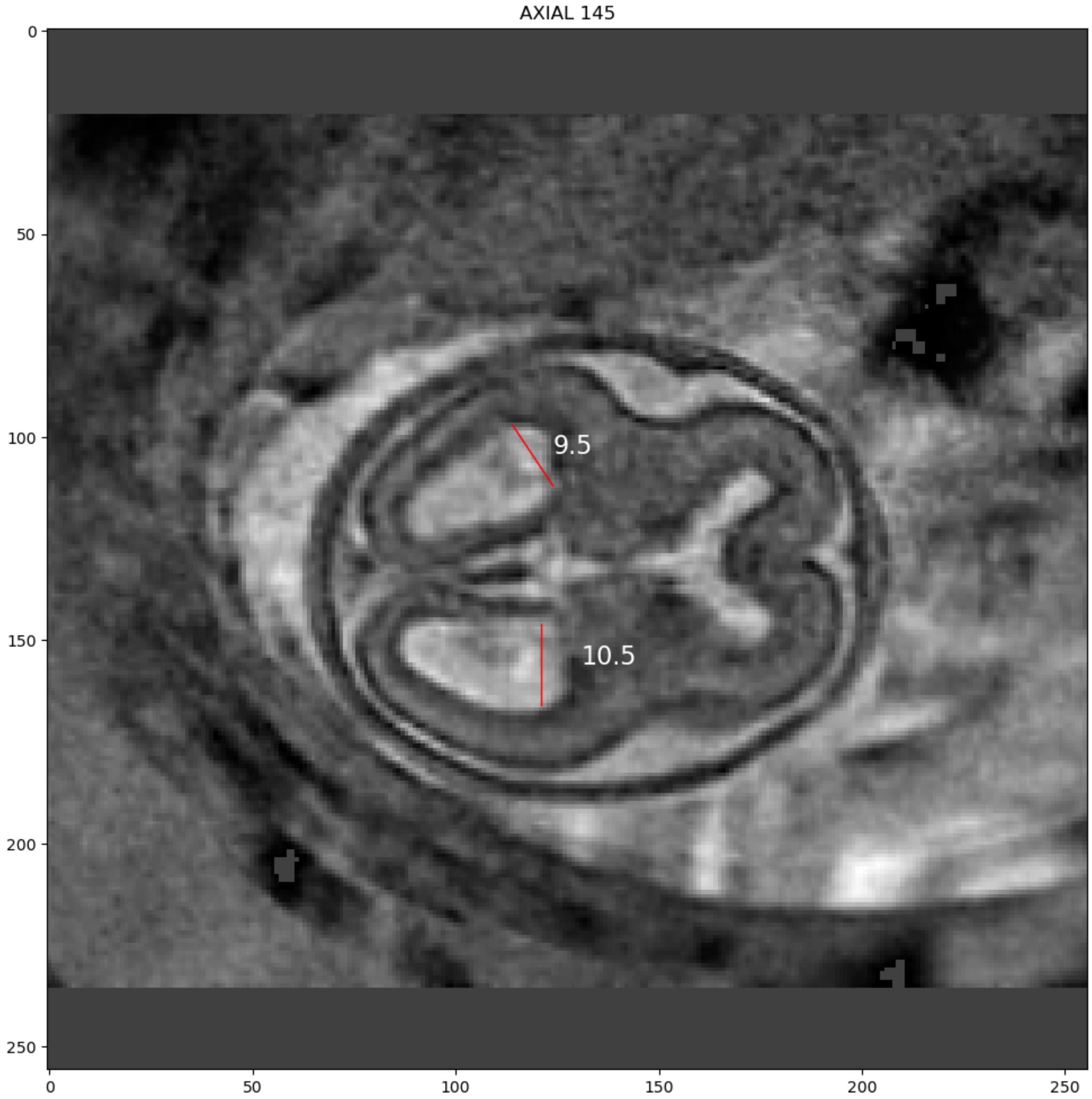


```
18 exam_000097_256.nii.gz
9.0 11.0 142
```

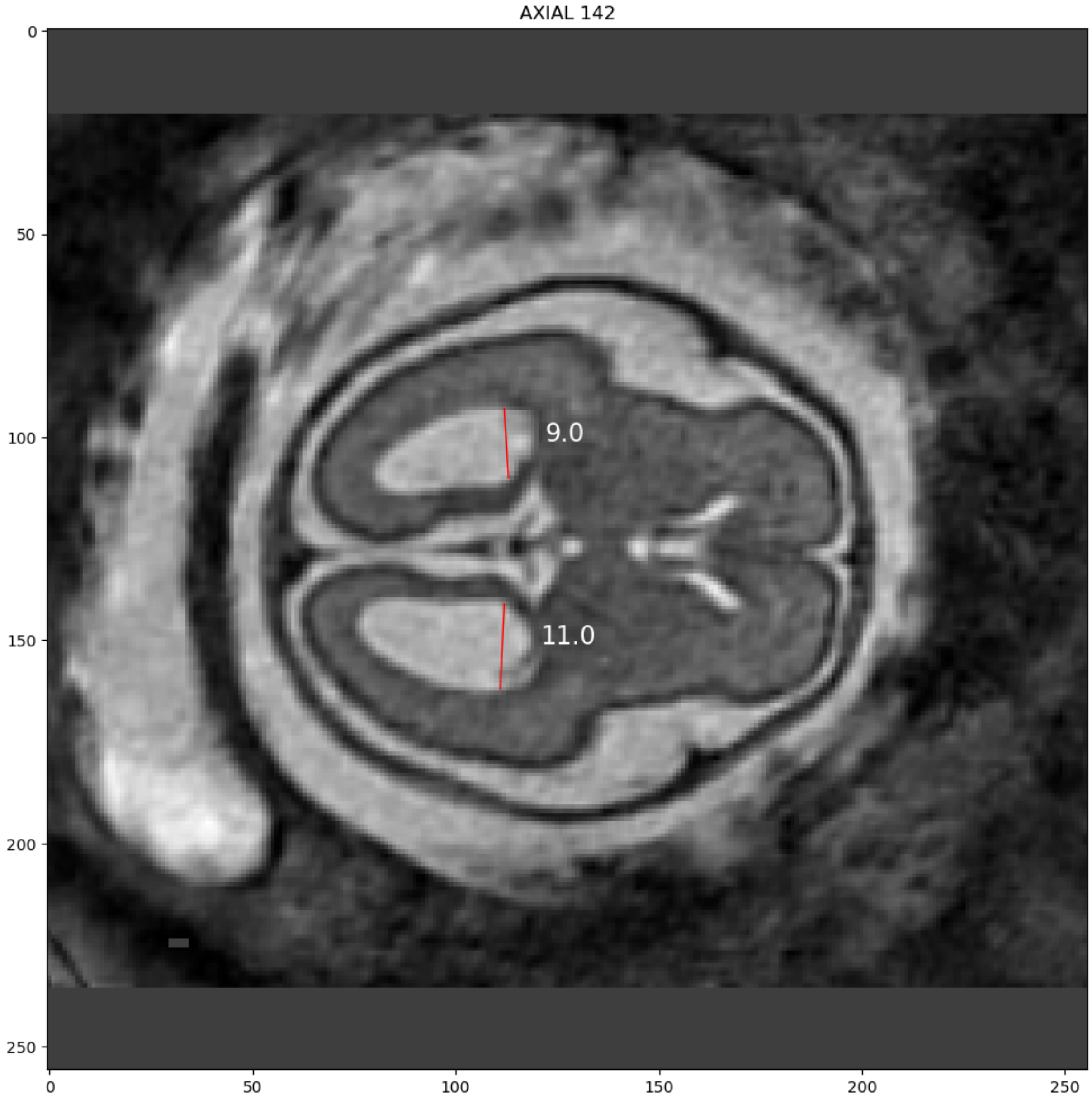


```
19 exam_000100_256.nii.gz
9.5 6.5 143
```

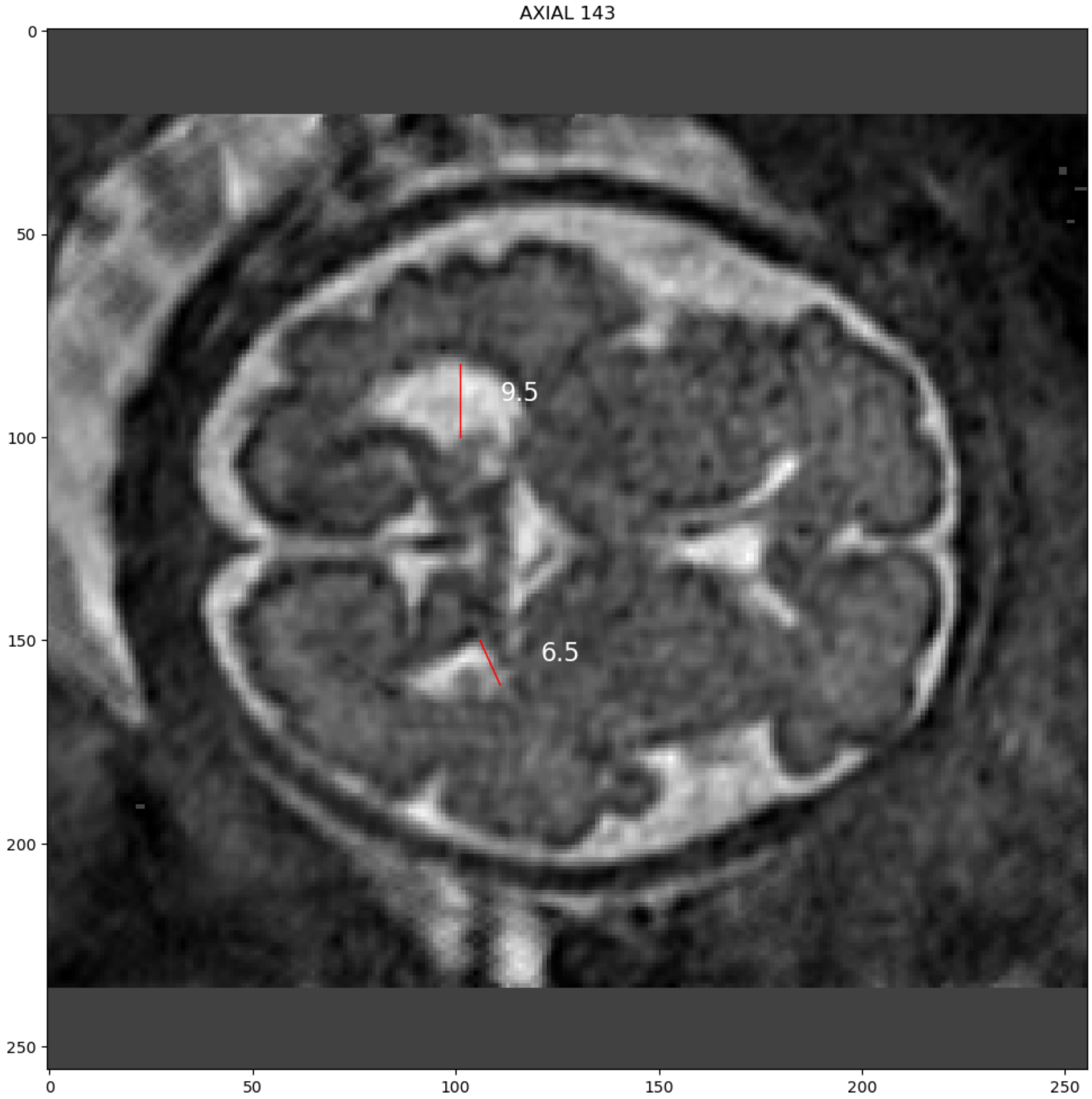


```
20 exam_000101_256.nii.gz
12.0 14.0 143
```

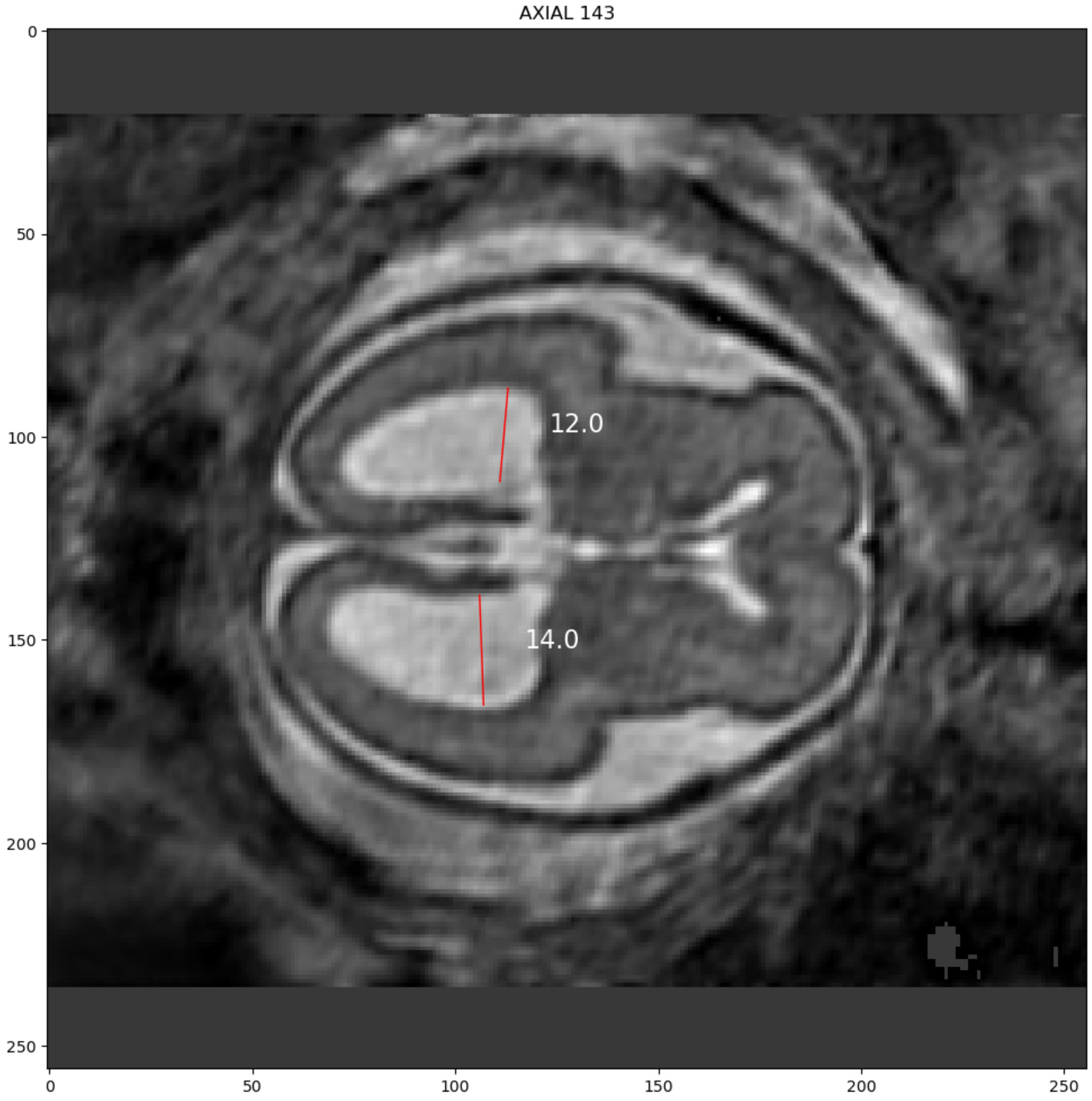


```
21 exam_000103_256.nii.gz
5.0 9.0 140
```

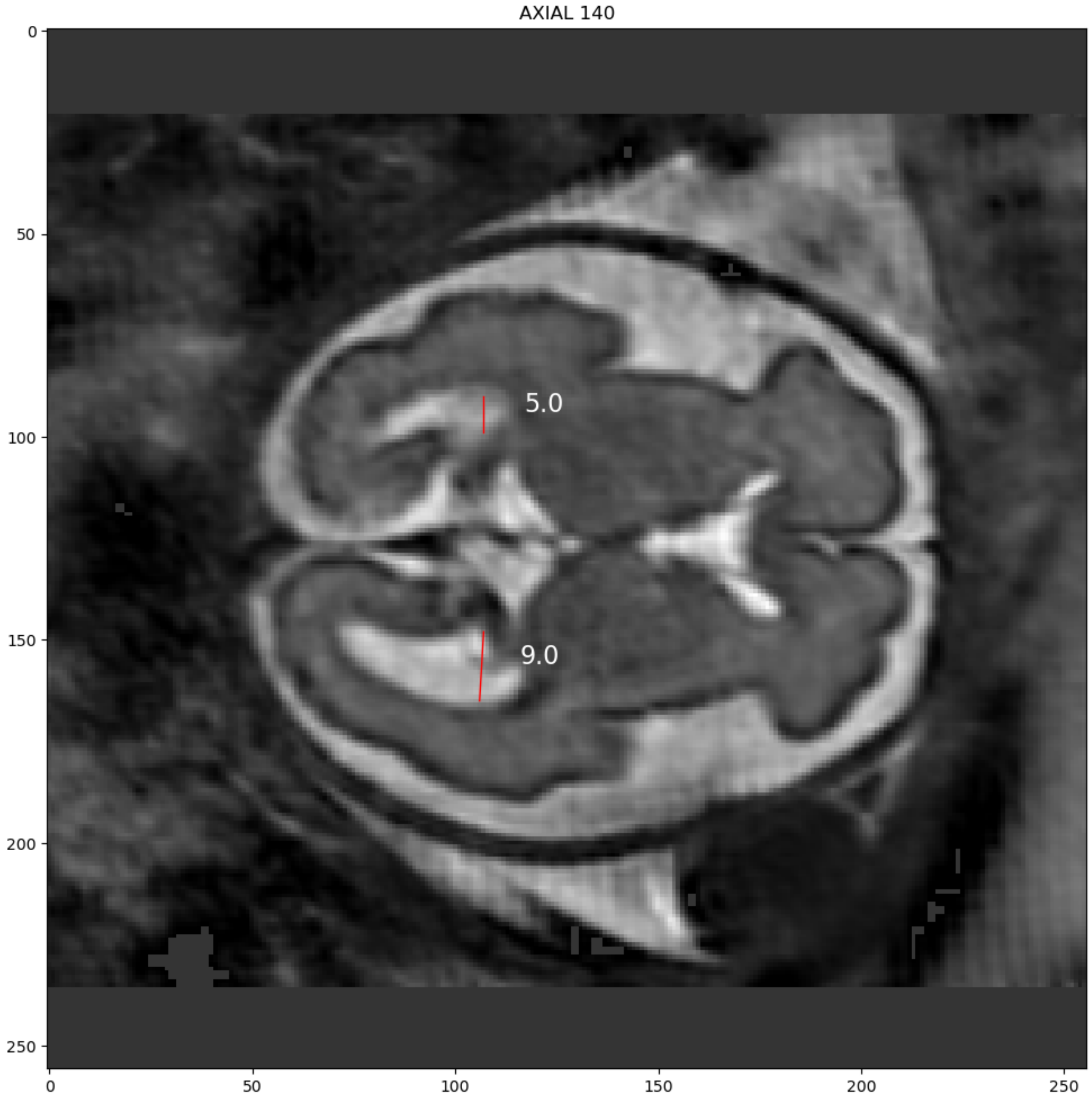

In [17]:
```
total_time = time.time() - total_start
print(f"Predict {len(test_dict)} cases cost: {total_time}s.")
```

```
Predict 22 cases cost: 33.99342393875122s.
```
